# Evolutionary genomics revealed interkingdom distribution of Tcn1-like chromodomain-containing Gypsy LTR retrotransposons among fungi and plants

**DOI:** 10.1186/1471-2164-11-231

**Published:** 2010-04-08

**Authors:** Olga Novikova, Georgiy Smyshlyaev, Alexander Blinov

**Affiliations:** 1 Laboratory of Molecular Genetic Systems, Institute of Cytology and Genetics, Novosibirsk, Russia; 2 Novosibirsk State University, Novosibirsk, Russia

## Abstract

**Background:**

Chromodomain-containing Gypsy LTR retrotransposons or chromoviruses are widely distributed among eukaryotes and have been found in plants, fungi and vertebrates. The previous comprehensive survey of chromoviruses from mosses (Bryophyta) suggested that genomes of non-seed plants contain the clade which is closely related to the retrotransposons from fungi. The origin, distribution and evolutionary history of this clade remained unclear mainly due to the absence of information concerning the diversity and distribution of LTR retrotransposons in other groups of non-seed plants as well as in fungal genomes.

**Results:**

In present study we preformed *in silico *analysis of chromodomain-containing LTR retrotransposons in 25 diverse fungi and a number of plant species including spikemoss *Selaginella moellendorffii *(Lycopodiophyta) coupled with an experimental survey of chromodomain-containing Gypsy LTR retrotransposons from diverse non-seed vascular plants (lycophytes, ferns, and horsetails). Our mining of Gypsy LTR retrotransposons in genomic sequences allowed identification of numerous families which have not been described previously in fungi. Two new well-supported clades, Galahad and Mordred, as well as several other previously unknown lineages of chromodomain-containing Gypsy LTR retrotransposons were described based on the results of PCR-mediated survey of LTR retrotransposon fragments from ferns, horsetails and lycophytes. It appeared that one of the clades, namely Tcn1 clade, was present in basidiomycetes and non-seed plants including mosses (Bryophyta) and lycophytes (genus *Selaginella*).

**Conclusions:**

The interkingdom distribution is not typical for chromodomain-containing LTR retrotransposons clades which are usually very specific for a particular taxonomic group. Tcn1-like LTR retrotransposons from fungi and non-seed plants demonstrated high similarity to each other which can be explained by strong selective constraints and the 'retained' genes theory or by horizontal transmission.

## Background

Retrotransposons are a class of mobile genetic elements, which use reverse transcription in their transposition. Five orders of retrotransposons are recognized: those having long terminal repeats (LTRs) (LTR retrotransposons); those lacking LTRs (non-LTR retrotransposons); DIRS retrotransposons; Penelope-like retrotransposable elements; and short interspersed nuclear elements (SINEs). According to the modern classification, LTR retrotransposons are divided into several superfamilies: Copia (Pseudoviridae), Gypsy (Metaviridae), Bel-Pao, Retrovirus (Retroviridae), and ERV [1].

Chromodomain-containing LTR retrotransposons or chromoviruses are the most widespread lineage of Gypsy LTR retrotransposons and are present in genomes of fungi as well as in plants and vertebrates [2,3]. The characteristic feature of chromoviruses is the presence of an additional domain - the chromodomain (CHD). CHDs are present in various eukaryotic proteins involved in chromatin remodeling and regulation of gene expression during development [4-6]. CHDs perform a wide range of diverse functions including chromatin targeting and proteinDNA/RNA interactions [6]. Recently, it has been shown that the CHDs target integration of new LTR retrotransposon copies into heterochromatin by recognizing histone modifications [7].

Our previous comprehensive survey of chromoviruses from mosses (Bryophyta) suggested that the diversity of CHD-containing Gypsy LTR retrotransposons in plant genomes is underestimated [8,9]. There are four wellknown CHD-containing Gypsy LTR retrotransposon clades widely distributed among gymnosperms and angiosperms: Tekay, CRM, Galadriel and Reina [2,3]. Four novel clades were found to be present in mosses. Moreover, we showed that representatives from one of the moss-specific clades are more closely related to retrotransposons from fungi than to retrotransposons from plants. Although we proposed that the retrotransposons from this clade could have been 'retained' from the last common ancestor of Fungi/Metazoa lineage and plants, the origin of this clade remains unclear.

The questions addressed in current investigation are as follows: (1) what kind of Gypsy LTR retrotransposons from fungi are closely related to the LTR retrotransposons detected in mosses; (2) how widely those clades which were previously identified in mosses are distributed among other non-seed plants including lycophytes, ferns and horsetails; and (3) what is the origin of the clade which is common for non-seed plants and fungi. The *in silico *analysis of Gypsy LTR retrotransposons in 25 species of fungi, genomes of which available in public databases, along with survey of related LTR retrotransposons from whole genome sequences (WGS) and expressed sequence tags (ESTs) of diverse plants including spikemoss *Selaginella moellendorffii *(Lycopodiophyta) and PCR-based screening of ferns, horsetails and lycophytes showed that a common clade of CHD-containing Gypsy LTR retrotransposons can be found in mosses, lycophytes from genus *Selaginella*, and basidiomycetes. According to the classification of CHD-containing Gypsy LTR retrotransposons proposed by Gorinsek et al. (2004) [3] this clade has name Tcn1. It seems that Tcn1 is a unique clade of chromoviruses which has a wide inter-kingdom distribution. Tcn1-like LTR retrotransposons from fungi and non-seed plants demonstrated higher similarity to each other in comparison with LTR retrotransposons from other clades. This can be explained by strong selective constraints and the 'retained' genes theory or by horizontal transmission.

## Results

### Gypsy LTR retrotransposons survey from fungal genomes

The Gypsy LTR retrotransposons mining from fungal genomes was initiated in an attempt to identify retrotransposons closely related to those which were found in mosses (Bryophyta) [8]. The survey was performed using genome sequence data for the 25 fungal species listed in Table 1. The hemiascomycetous yeasts were not included in the present investigation since a comprehensive survey of LTR retrotransposons from this group of ascomycetes was recently published [10]. First, reverse transcriptase (RT) and integrase (Int) coding regions of Gypsy LTR retrotransposons were detected in genomic sequences using algorithm based on hidden Markov model implemented in uGENE software http://ugene.unipro.ru/. The transposable elements thus identified were then classified into families based on RT and Int domains sequence similarity. Members of the same family shared high amino acid identity (90-100%) but had very little similarity to elements from other families. Our survey has identified more than 150 novel Gypsy LTR retrotransposon families which have not been described previously (Additional file 1).

**Table 1:** List of fungal species, genomes of which were analyzed *in silico *in present study

Phylum/Subphylum	Class	Species and strain	genome size (Mb)	LTR^a^
Ascomycota/Pezizomycotina	Sordariomycetes	*Chaetomium globosum *CBS 148.51	36	23

		*Fusarium oxysporum *4286 FGSC	60	39

		*Fusarium verticillioides *7600	46	4

		*Nectria haematococca *MPVI	40	65

		*Podospora anserina *S mat+	37	11

		*Trichoderma reesei *QM6a	33	5

		*Trichoderma virens *Gv29-8	38	5

	Eurotiomycetes	*Aspergillus clavatus *NRRL 1	35	29

		*Aspergillus niger *ATCC1015	37	1

		*Aspergillus terreus *NIH2624	35	3

		*Coccidioides immitis *RS	29	236

		*Histoplasma capsulatum *NAm1	28	74

		*Uncinocarpus reesii *1704	30	64

	Leotiomycetes	*Sclerotinia sclerotiorum *1980	38	25

		*Botrytis cinerea *B05.10	38	25

	Dothideomycetes	*Alternaria brassicicola *ATCC 96866	30	108

		*Pyrenophora tritici-repentis *Pt-1C-BFP	37.8	118

		*Stagonospora nodorum *SN15	37	12

Basidiomycota/Agaricomycotina	Agaricomycetes (Homobasidiomycetes)	*Amanita bisporigera*	58	7

		*Coprinus cinereus *Okayama7#130	38	160

		*Laccaria bicolor *S238N	61	114

		*Postia placenta *MAD-698	90	921

Basidiomycota/Pucciniomycotina	Urediniomycetes	*Sporobolomyces roseus*	21	6

		*Puccinia graminis f. sp. tritici*	81.5	534

Chytridiomycota	Chytridiomycetes	*Batrachochytrium dendrobatidis *JEL423	24	1

^a ^total copy number of detected Gypsy LTR retrotransposons

**Figure 1:**
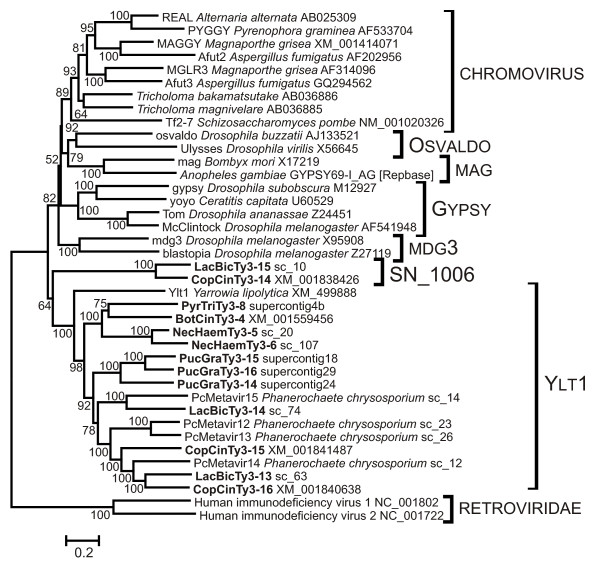
**Neighbor-joining (NJ) phylogenetic trees based on RT and partial Int amino acid sequences of Gypsy LTR belonging to Ylt1 and SN_1006 clades**. Statistical support was evaluated by bootstrapping (1000 replications); nodes with bootstrap values over 50% are indicated. The Gypsy LTR retrotransposons clades are shown on the right and include Chromovirus, Osvaldo, mag, Gypsy, mdg3, SN_1006 and Ylt1. Sequences of human immunodeficiency viruses (Retroviridae) were used as outgroup. The name of the host species and accession number are indicated for all elements taken from GenBank. Newly identified retrotransposons are highlighted by bold; localization in genomic sequence is indicated for each of them. Genomic sequences of *Laccaria bicolor *S238N and *Nectria haematococca *MPVI have been taken from The DOE Joint Genome Institute [55]; the following species are available at Broad Institute [54]: *Botrytis cinerea *B05.10; *Pyrenophora tritici-repentis *Pt-1C-BFP; *Coprinus cinereus *okayama7#130; *Puccinia graminis f. sp. tritici*. For more details: Additional files 1, 5 and 6.

The newly identified Gypsy LTR retrotransposons fall into two major, distinct lineages according to phylogenetic analysis based on RT and partial Int domains: chromoviruses (or CHD-containing Gypsy LTR retrotransposons) and Ylt1-like LTR retrotransposons. Two retrotransposons (LacBicTy3-15 from *Laccaria bicolor *and CopConTy3-14 from *Coprinus cinereus*) formed their own lineage closely related to Ylt1-like LTR retrotransposons. This lineage cannot be attributed to Ylt1 because of low bootstrap support (only 64%; Figure 1) and was named SN_1006. Ylt1-like LTR retrotransposons were found in a number of fungal species (Figure 1). Previously, Ylt1-like LTR retrotransposons were reported for *Yarrowia lipolytica *(original Ylt1 retrotransposon) [11], *Candida albicans *(Tca3 element) [12], and basidiomycete *Cryptococcus neoformans *[13]. In the present study, LTR retrotransposons belonging to the Ylt1 lineage have been found in both ascomycete and basidiomycete fungi. They fall into several clearly separated groups: the branch formed by transposable elements from Basidiomycota, the group of retrotransposons from ascomycetes, and the branch of the original Ylt1 LTR retrotransposon (Figure 1).

**Figure 2:**
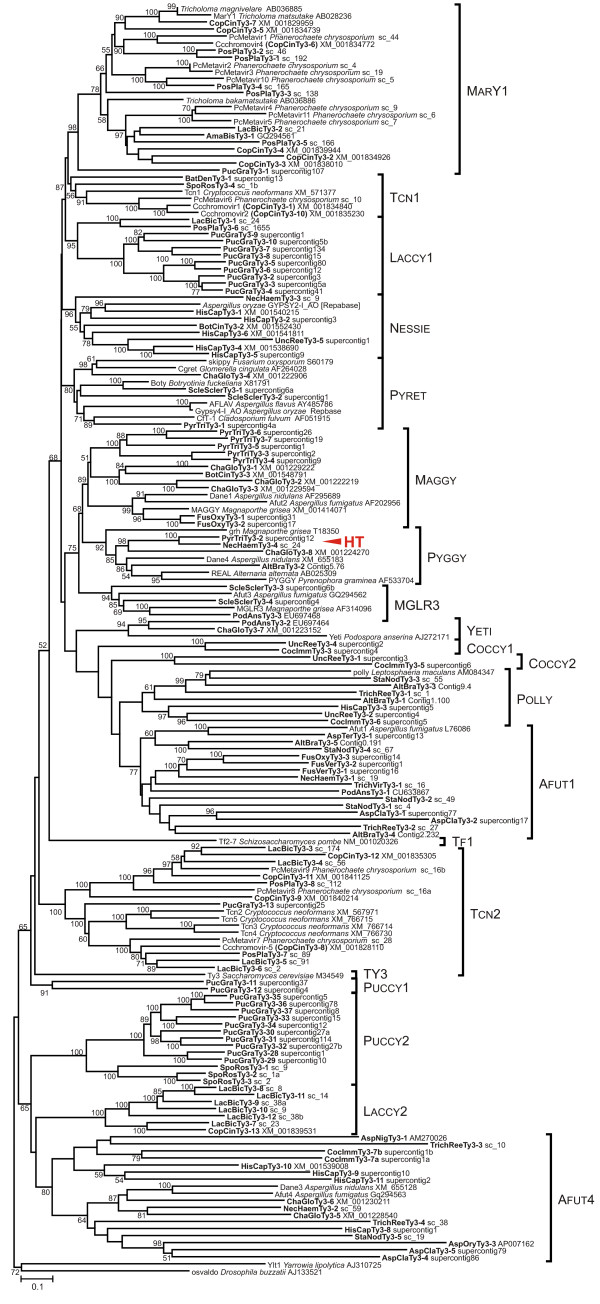
**Neighbor-joining (NJ) phylogenetic trees based on RT and partial Int amino acid sequences of Gypsy LTR retrotransposons including newly described fungal chromodomain-containing LTR retrotransposons**. Statistical support was evaluated by bootstrapping (1000 replications); nodes with bootstrap values over 50% are indicated. The clades are shown on the right. The name of the host species and accession number are indicated for all elements taken from GenBank. Newly identified retrotransposons are highlighted in bold; localization in genomic sequence is indicated for each of them. Genomic sequences of *Trichoderma reesei *QM6a, *Trichoderma virens *Gv29-8, *Nectria haematococca *MPVI, *Aspergillus niger *ATCC1015, *Alternaria brassicicola *ATCC 96866, *Stagonospora nodorum *SN15, *Laccaria bicolor *S238N, *Postia placenta *MAD-698, and *Sporobolomyces roseus *have been taken from The DOE Joint Genome Institute [55]; the following species are available at Broad Institute [54]: *Chaetomium globosum *CBS 148.51; *Fusarium oxysporum *4286 FGSC;*Fusarium verticillioides *7600; *Aspergillus clavatus *NRRL 1; *Aspergillus terreus *NIH2624; *Coccidioides immitis *RS; *Histoplasma capsulatum *NAm1; *Uncinocarpus reesii *1704; *Sclerotinia sclerotiorum *1980; *Botrytis cinerea *B05.10; *Pyrenophora tritici-repentis *Pt-1C-BFP; *Coprinus cinereus *okayama7#130; *Puccinia graminis f. sp. tritici*; *Batrachochytrium dendrobatidis *JEL423. The possible horizontal transmission (HT) is marked. For more details: Additional files 1, 5 and 6.

Twenty monophyletic clades can be recognized in the phylogenetic tree of fungal CHD-containing Gypsy LTR retrotransposons, nine of which have been previously reported [3]. Thirteen clades are specific for ascomycetes (Nessie, Pyret, Maggy, Pyggy, MGLR3, Yeti, Coccy1, Coccy2, Polly, Afut1, Tf1, Ty3, and Afut4), six have been found only in genomes of basidiomycetes (MarY1, Laccy1, Laccy2, Tcn2, Puccy1, and Puccy2) and one clade (Tcn1) is present in both basidiomycetes and chytridiomycetes (*Batrachochytrium dendrobatidis *JEL423) (Figure 2).

**Figure 3:**
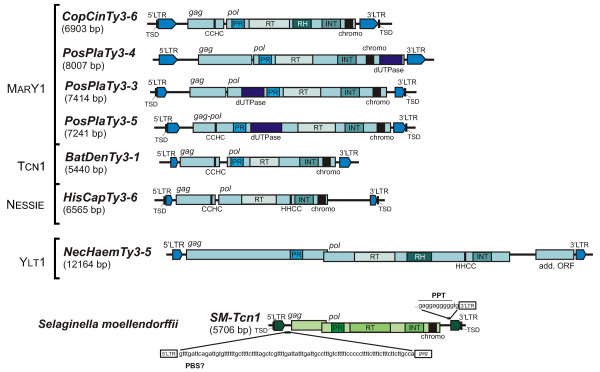
**Structural organization of a number of full-length LTR retrotransposons from fungi and SM-Tcn1 LTR retrotransposon from spikemoss *Selaginella moellendorffii *identified in present study**. The clade for each of elements is shown on the left. Abbreviations: LTR - long terminal repeat, TSD - target site duplication, PR - proteinase, RT - reverse transcriptase, RH - ribonuclease H, Int - core integrase, chromo - chromodomain, dUTPase - deoxyuridine triphosphatase domain, CCHC and HHCC - Zn-finger motifs, add. ORF - additional open reading frame with unknown function, PPT - polypurine tract, PBS? - no putative primer-binding site was found for SM-Tcn1.

For each of the newly identified retrotransposon families, we attempted to isolate a full-length representative or to reconstruct it using overlaps between partial sequences. The lengths of the elements thus identified varied greatly from approximately 4.4 kb to more than 13 kb. The structural features for each family are listed in Additional Table S1 (Additional file 1). The majority of full-length LTR retrotransposons had either a single open reading frame (ORF) encoding a fused Gag-Pol polyprotein or two ORFs encoding separate proteins (Figure 3). The Gag protein sequences differed greatly between families. Nevertheless, cysteine motifs characterized by the amino acid sequence C-X_2_-C-X_4_-H-X_4_-C (CCHC) were found in Gag for some of the identified Gypsy LTR retrotransposons (see Additional file 1). The Pol polyproteins sequences were more conserved than Gag, especially with the RT and Int domains. RT, Int, PR (proteinase) and chromodomains (CHDs, in chromoviruses) were detected. Characteristic motifs were found throughout the Gag and Pol sequences of all of the putative intact element copies.

In addition to the abovelisted enzymatic domains, a deoxyuridine triphosphatase domain (dUTPase) has been found in several LTR retrotransposons from the basidiomycete *Postia placenta *MAD-698. The location of this domain varied among diverse families of PosPlaTy3 elements. It can be found either at amino-terminus (PosPlaTy3-3) and carboxyl-terminus of Pol (PosPlaTy3-4) or between PR and RT domains (PosPlaTy3-5) (Figure 3). The presence of dUTPase in LTR retrotransposon sequences has been described earlier for the elements from a basidiomycete *Phanerochaete chrysosporium *and an ascomycete *Tuber melanosporum *[14,15]. The role and origin of this domain remained unclear. Moreover, it seems that the described LTR retrotransposons acquire this domain independently from different sources (Additional file 2) [14]. It was assumed that the presence of dUTPase allows viruses that contain this domain to replicate in non-dividing cells, in which cellular dUTPase activity is absent because replication of DNA does not occur [16].

### Tnc1 clade is found in non-seed plants

**Figure 4:**
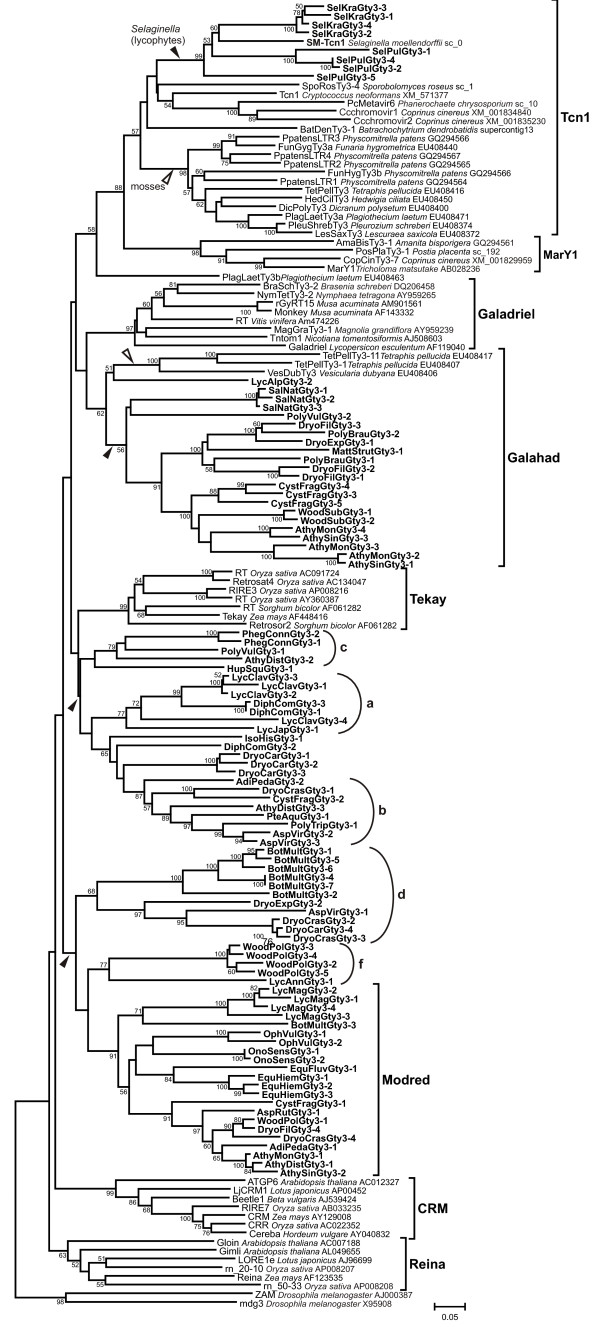
**Neighbor-joining (NJ) phylogenetic tree based on RT nucleotide sequences of CHD-containing Gypsy LTR retrotransposons including newly described elements from monilophytes and lycophytes plants (highlighted in bold)**. Statistical support was evaluated by bootstrapping (1000 replications); nodes with bootstrap values over 50% are indicated. The name of the host species and accession number are indicated for LTR retrotransposons taken from GenBank. Four diverse clusters of LTR retrotransposons from mosses, monilophytes and lycophytes are shown by arrows. The group of Tcn1-like LTR retrotransposons from mosses (Bryophyta) is also indicated. Previously known clades, clades described in this study, and unclassified lineages (a-f) are shown on the right.

Further phylogenetic analysis revealed that previously described CHD-containing LTR retrotransposons from mosses including PpatensLTR retrotransposons isolated from genomic sequence of moss *Physcomitrella patens *formed a common branch with Tcn1-like LTR retrotransposons from fungi (Figure 4) [8]. In an attempt to determine the distribution of the Tcn1clade among plants, we used public databases for further survey of LTR retrotransposons including genomic databases for red and green algae, spikemoss *Selaginella moellendorffii*, and seed plants (see Materials and Methods section). A Tcn1-like LTR retrotransposon search was implemented with BLAST (blastp and blastx). Amino acid sequences of RT and Int domains of known Tcn1-like (Tcn1 from *C. neoformans*, Ccchromovir1 and Ccchromovir2 from *C. cinereus*, PcMetavir6 from *Phanerochaete chrysosporium*, and PpatensLTRs from *P. patens*) and newly identified retrotransposons (SpoRosTy3-4 and BatDenTy3-1) were used as the queries. The Tcn1-like LTR retrotransposons were identified only in the whole genomic sequence of *Selaginella moellendorffii *(SM-Tcn1, Figure 4); none of the tested algae or seed plant genomes contained LTR retrotransposons from this clade. It seems that the Tcn1 clade can be found in basidiomycetes and chytridiomycetes fungi as well as non-seed plants (Bryophyta and Lycopodiophyta).

The whole sequence of SM-Tcn1 LTR retrotransposon was obtained from WGS. SM-Tcn1 is 5704 bp in length and carries two putative ORFs. ORF1 or *gag *(969 bp in length) encodes a 323 amino acid (aa) protein with strong similarity to retroviral Gag proteins (pfam03732). ORF2 or *pol *(3714 bp) encodes a 1238 aa polyprotein, with characteristic retroviral aspartyl protease (PR), RT, Int, and CHD domains (Figure 3; Additional file 3). SM-Tcn1 possesses 440 bp LTRs with conserved features, including the dinucleotide end sequences (TG...CA). Target site duplication (AACAC...AACAC) was also detected for the described copy of SM-Tcn1. The LTRs contained TATA and CAAT boxes. No putative primer-binding site (PBS) was found. The PBS is necessary for initiation of reverse transcription and synthesis of the first strand complementary 5'LTR sequence [17]. Typical PBS is located near the 5'LTR and complementary to the 3' terminal nucleotides of the primer tRNA used for initiation. Other known mechanism for initiation of the first strand synthesis is self-priming, in this case a sequence derived from LTR is located just downstream of the 5'LTR [18,19]. However, evidences were found neither for tRNA priming nor for self-priming of SM-Tcn1 LTR retrotransposon. The sequence presented between 5' LTR and *gag *is conservative and thymine-rich (Figure 3). The possible mechanism for initiation of reverse transcription of SM-Tcn1 remained unclear. A polypurine tract (PPT) was detected immediately upstream of the 3'LTR. The PPT sequence is involved in second-strand DNA synthesis. The BLAST search (blastn) of full-length SM-Tcn1 retrotransposon indicated the presence of more than 200 hits in the *S. moellendorffii *genome. The close examination of identified copies showed that they have in average 91.7% nucleotide identity with the original SM-Tcn1 sequence. A BLAST search (tblastn) using SM-Tcn1 putative Pol protein as a query yielded more than 500 hits. The size of *S. moellendorffii *genome is only ~100 Mbp [20], thus SM-Tcn1 retrotransposons comprise ca. 1.5% of genomic sequence.

### Gypsy LTR retrotransposons from non-seed vascular plants

The bioinformatic survey of CHD-containing Gypsy LTR retrotransposons, which allowed us to identify Tcn1-like retrotransposons in a few basidiomycetes, *P. patens *and *S. moellendorffii*, did not provide a satisfactory answer to the question concerning distribution of this clade among non-seed vascular plants. Therefore, we used PCR with degenerate primers to investigate the distribution of CHD-containing Gypsy LTR retrotransposons in 26 ferns and horsetails (monilophytes) belonging to three classes: Psilotopsida, Polypodiopsida and Equisetopsida, and in 10 lycophytes from two classes: Isoetopsida and Lycopodiopsida (Table 2).

**Table 2:** List of monilophytes and lycophytes, which were analyzed experimentally in present study

Division	Class	Family	Species	Chr (Athila)^a^
Moniliformopses	Psilotopsida	Ophioglossaceae	*Ophioglossum vulgatum *L.	2

			*Botrychium multifidum *(Gmelin) Rupr.	6

	Polypodiopsida	Dennstaedtiaceae	*Pteridium aquilinum *(L.) Kuhn	1 (1)

		Pteridaceae	*Adiantum pedatum *L.	2

		Aspleniaceae	*Asplenium viride *Huds.	3 (1)

			*Asplenium ruta-muraria *L.	1

		Woodsiaceae	*Athyrium sinense *Rupr.	3 (3)

			*Athyrium distentifolium *Tausch ex Opiz	3 (1)

			*Athyrium monomachii *(Kom.) Kom.	4 (1)

			*Cystopteris fragilis *(L.) Bern.	5

			*Woodsia polystichoides *DC Eaton	5

			*Woodsia subcordata *Turcz.	2

		Thelypteridaceae	*Phegopteris connectilis *(Michx.) Watt.	2

		Onocleaceae	*Matteuccia struthiopteris *(L.) Tod.	1 (1)

			*Onoclea sensibilis *L.	2

		Dryopteridaceae	*Dryopteris expansa *(Presl) Fraser-Jenk. & Jermy	2

			*Dryopteris filix-mas *(L.) Schott	4

			*Dryopteris crassirhizoma *Nak.	4

			*Dryopteris carthusiana *(Vill.) Fuchs	4

			*Polystichum braunii *(Spenner) Fée	2

			*Polystichum tripteron *(Kunze) Presl	1

		Polypodiaceae	*Polypodium vulgare *L.	2

			*Pyrrosia lingua *(Thunb.) Farw.	-- (1)

		Salviniaceae	*Salvinia natans *(L.) All.	3

	Equisetopsida	Equisetaceae	*Equisetum hiemale *L.	3

			*Equisetum fluviatile *L.	1

Lycopodiophyta	Isoetopsida	Isoetaceae	*Isoetes histrix *Bory	1

		Selaginellaceae	*Selaginella kraussiana *(Kunze) A. Braun	4

			*Selaginella pulvinata *(Hook. & Grev.) Maxim.	4

	Lycopodiopsida	Lycopodiaceae	*Diphasiastrum complanatum *(L.) Holub	3

			*Huperzia squarrosa *(Forst.) Trevis.	1

			*Lycopodium alpinum *L.	1

			*Lycopodium clavatum *L.	4

			*Lycopodium magellanicum *Hert. ex Nessel	4

			*Lycopodium japonicum *Thunb. ex Murr.	1

			*Lycopodium annotinum *L.	1 (1)

^a ^number of unique sequences for CHD-containing LTR retrotransposons (Chr) and Athila-like LTR retrotransposons obtained in present study

The estimated diversity of monilophytes (= Infradivision Moniliformopses) is about 9000 species and includes horsetails, whisk ferns, and all eusporangiate and leptosporangiate ferns [19]. Most of the species examined in the present study were leptosporangiate ferns from the order Polypodiales, class Polypodiopsida. This order covers more than 80% of current known diversity of ferns [21]. Additionally, one representative of heterosporous ferns, *Salvinia natans *(Polypodiopsida, Salviniales), two ophioglossoid ferns (Psilotopsida, Ophioglossales) and three horsetails (Equisetopsida, Equisetales) were included [22]. Lycophytes are much less diverse in comparison with monilophytes and comprise less than 1% of extant land plants (around 1200 living species). Three major lineages are distinguished among lycophytes: clubmosses and firmosses (Lycopodiaceae), spikemosses (Selaginellaceae), and quillworts (Isoetaceae) [23]. Among lycophytes included in the present study are *Isoetes *and *Huperzia *species (Isoetaceae) as well as two *Selaginella *species (Selaginellaceae), which belong to the class Isoetopsida, and seven diverse species from Lycopodiaceae (Lycopodiales, Lycopodiopsida).

The presence of CHD-containing Gypsy LTR retroelements among the listed plants was tested by amplifying genomic DNA with previously developed degenerate oligonucleotide primers [8,24]. Consistent with the spacing of reverse transcriptase (RT) domains, the amplified PCR products were approximately 320 bp in length. In total, 98 clones with sequence similarity to known RT sequences were isolated, of which 76 were from monilophytes and 22 from lycophytes. The preliminary blastp search revealed that 10 clones were not from CHDcontaining LTR retrotransposons but were from Athila-like Gypsy elements (Additional file 4). Many representatives of this clade possess not only classical gag and pol sequences, but also an additional open reading frame that might encode an env-like protein [25,26].

The phylogenetic relationships among obtained clones and known CHD-containing Gypsy LTR retrotransposons, extracted from databases, were reconstructed using neighbor-joining (NJ) analysis based on the multiple alignment of nucleotide sequences of RT fragment (Figure 4). The Gypsy LTR retrotransposons from *Drosophila melanogaster *were used as an outgroup. The newlyidentified LTR retrotransposon grouped into four large clusters on the phylogenetic tree. The group of clones from diverse monilophytes and one LTR retrotransposon from lycophytes *Lycopodium alpinum *(LycAlpTy3-1 clone) form a common group with previously described retroelements from mosses *Tetraphis pellucida *and *Vesicularia dubyana *[8]. Although the bootstrap support is below 50% (data not shown), this new clade (named "Galahad") seems to be a sister group to the Galadriel clade. Galahad appears to be one of the oldest widely distributed clades of CHD-containing LTR retrotransposons from plants. Since Galahad clade was found in all non-seed plants including mosses, the probable age of this clade would be in the range of 400-700 Myr, which is estimated time divergence of liverworts and mosses from vascular plants [27].

The second group is formed by LTR retrotransposons from both monilophytes and lycophytes. The phylogenetic analysis did not provide support for a monophyletic origin of this cluster. Moreover, the relationships inside the cluster remained unclear, with the exception of several lineages. One of the lineages ('a' in Figure 4), contained members from lycophytes in the family Lycopodiaceae: *Lycopodium clavatum *(LycClavGty3 clones), *L. japonicum *(LycJapGty3-1 clone), and *Diphasiastrum complanatum *(DiphComGty3 clones). Another lineage seems to have had a long-term association with fern genomes, since it appears to be widely distributed among leptosporangiate ferns and can be found in Dennstaedtiaceae (*Pteridium aquilinum*), Pteridaceae (*Adiantum pedatum*), Aspleniaceae (*Asplenium viride*), Woodsiaceae (*Athyrium distentifolium *and *Cystopteris fragilis*), and Dryopteridaceae (*Dryopteris crassirhizoma *and *Polystichum tripteron*) (lineage 'b' on Figure 4). Additionally, five satellite lineages, represented mostly by single clones can be found on the phylogenetic tree.

The largest group is represented by 37 LTR retrotransposons. Three clearly separated clusters can be found inside this group (marked as 'd', 'f', and Mordred on Figure 4). One of these clusters is formed by LTR retrotransposons from Ophioglossaceae (*Botrychium multifidum*), Aspleniaceae (*A. viride*) and Dryopteridaceae (*Dryopteris expansa*, *D. carthusiana*, and *D. crassirhizoma*). The second cluster has a bootstrap support of 77% and is represented by clones isolated from *Lycopodium annotinum *(Lycopodiaceae) and *Woodsia polystichoides *(Woodsiaceae). The last monophyletic cluster, clade Mordred, is the largest, well-supported clade which is widely distributed among representatives of all investigated classes except Isoetopsida. It was found to be present in fern genomes from families Ophioglossaceae (*Ophioglossum vulgatum *and *Botrychium multifidum*), Pteridaceae (*Adiantum pedatum*), Woodsiaceae (*Athyrium sinense*, *A. distentifolium*, *A. monomachii*, *C. fragilis*, and *W. polystichoides*), Onocleaceae (*Onoclea sensibilis*), and Dryopteridaceae (*Dryopteris filix-mas *and *D. crassirhizoma*); in horsetails *Equisetum hiemale *and *E. fluviatile *(Equisetaceae); and lycophyte *Lycopodium magellanicum *(Lycopodiaceae).

The Tcn1-like LTR retrotransposons were detected only in *Selaginella *species, *S. kraussiana *(SelKraGty3 clones) and *S. pulvinata *(SelPulGty3 clones) in addition to the previously described LTR retrotransposons from mosses and SM-Tcn1 LTR retrotransposon from *Selaginella moellendorffii *(Figure 4) [8]. The absence of Tcn1-like clones isolated from other lycophytes, ferns, and horsetails can be explained by failed PCR amplification due to the high divergence of Tcn1-like elements in genomes of these species or, more likely, by lack of these elements from their genomes.

### Tcn1-like LTR retrotransposons: 'retained' or horizontally transmitted?

As a rule chromoviruses clades are specific for a particular group of eukaryotic organisms such as Ascomycota fungi (Nessie, Pyret, Maggy, Pyggy, MGLR3, Yeti, Coccy1, Coccy2, Polly, Afut1, Tf1, Ty3, and Afut4), Basidiomycota fungi (MarY1, Laccy1, Laccy2, Tcn2, Puccy1, and Puccy2), or plants (Reina, CRM, Tekay, Galadriel, and Chlamyvir as well as additional less investigated clades from mosses) [3,8]. In the light of such specificity, it was unexpected to find a clade containing elements from basidiomycetes and non-seed plants. Nevertheless, it seems that Tcn1 clade has an interkingdom distribution and can be found in a number of fungi, diverse mosses (Bryophyta) as well as in lycophytes (genus *Selaginella*). Such a wide distribution makes the Tcn1 clade unique among the CHD-containing Gypsy LTR retrotransposons. The interkingdom distribution of Tcn1 clade could be the result of horizontal transmission (HT) of LTR retrotransposons among fungi and plants; otherwise, Tcn1-like LTR retrotransposons could have been 'retained' by mosses and lycophytes from the most recent common ancestor of plants and Fungi/Metazoa lineage of eukaryotes [8,28].

The hypothesis of 'retained' genes is based on the observation that EST data of *Physcomitrella *contained a fraction of transcripts derived from putative genes ('retained' genes), which are not present in seed plants but can be found in other kingdoms including fungi. It was proposed that such retained genes along with *Physcomitrella*-specific (or moss-specific) genes encode functions that make mosses unique in terms of physiology and metabolism [28]. We used these data and compared the levels of similarity for RT-Int fragments from Tcn1-like LTR retrotransposons and two putatively retained genes from *Physcomitrella*, which showed a high similarity with functional genes from fungi: uric acid-xanthine permease (uapA, TIGR00801) and inorganic phosphate transporter (Pho88, pfam10032).

The pairwise comparisons between hypothetical Pho88 proteins from basidiomycetes *Coprinus cinereus *Okayama7#130, *Phanerochaete chrysosporium *and *Cryptococcus neoformans *revealed 51.3% to 64.9% similarity whereas only 23.0% identical amino acid residues was found on average in pairwise comparisons between fungal proteins and putative Pho88 from *Physcomitrella *(Table 3). The most closely related homolog for putative Pho88 from *P. patens *was found in *Schizosaccharomyces pombe *(26.8% of similarity). The similarity between uapA from *Physcomitrella *and *Cryptococcus *(36.9%) was almost the same as between proteins from *Cryptococcus *and *Coprinus *(39.6%) or *Cryptococcus *and *Phanerochaete *(43.2%). More then 67% of amino acid residues are identical in permeases from *Coprinus *and *Phanerochaete*. Predicted uapA from *Ashbya gossypii *and *Physcomitrella *share 40.7% of amino acid residues.

**Table 3:** Amino acid divergences of proteins and RT-Int fragments of CHD-containing Gypsy LTR retrotransposons from Tcn1, Pyggy and Pyret clades

Genes or LTR retrotransposons	Length	Amino acid identity (%)	Evolutionary rate (10^-9^)^b^
inorganic phosphate transporter (Pho88)^a^			

*Physcomitrella patens *(XM_001783642)/*Schizosaccharomyces pombe *(NM_001019478)	154 aa	26.8	0.420

*P. patens *(XM_001783642)/*Coprinus cinereus *(XM_001836748)	151 aa	23.1	0.445

*P. patens*/*Phanerochaete chrysosporium *(JGI: scaffold_7 [325337..326089])	151 aa	25.0	0.428

*P. patens*/*Cryptococcus neoformans *(XM_569621)	152 aa	20.1	0.521

*C. cinereus*/*Ph. chrysosporium*	151 aa	64.9	ND

*C. cinereus*/*C. neoformans*	151 aa	51.3	0.472

*Ph. chrysosporium*/*C. neoformans*	151 aa	58.6	ND

uric acid-xanthine permease (uapA)^a^			

*P. patens *(XM_001784081)/*Ashbya gossypii *(NM_212305)	466 aa	40.7	0.266

*P. patens*/*C. cinereus *(XM_001839036)	456 aa	38.4	0.288

*P. patens*/*Ph. chrysosporium *(JGI: scaffold_22 [347275..348820])	391 aa	43.6	0.263

*P. patens*/*C. neoformans *(AF542528)	472 aa	36.9	0.318

*C. cinereus*/*Ph. chrysosporium*	391 aa	67.9	ND

*C. cinereus*/*C. neoformans*	456 aa	39.6	0.560

*Ph. chrysosporium*/*C. neoformans*	391 aa	43.2	ND

Tcn1			

Tcn1 *C. neoformans*/Ccchromovir-1 *C. cinereus*	684 aa	53.6	0.441

Tcn1 *C. neoformans*/PcMetavir6 *Ph. chrysosporium*	684 aa	52.2	ND

Tcn1 *C. neoformans*/BatDenTy3-1 *B. dendrobatidis*	677 aa	49.1	0.381

PcMetavir6 *Ph. chrysosporium*/Ccchromovir-1 *C. cinereus*	688 aa	72.4	ND

PcMetavir6 *Ph. chrysosporium*/BatDenTy3-1 *Batrachochytrium dendrobatidis*	677 aa	47.0	0.412

BatDenTy3-1 *B. dendrobatidis*/Ccchromovir-1 *C. cinereus*	677 aa	46.5	0.411

Tcn1 *C. neoformans*/PpatensLTR1 *P. patens*	675 aa	52.0	0.216 (HT)

Tcn1 *C. neoformans*/SM-Tcn1 *Selaginella moellendorffii*	682 aa	50.3	0.235 (HT)

PcMetavir6 *Ph. chrysosporium*/PpatensLTR1 *P. patens*	675 aa	51.8	0.217 (HT)

PcMetavir6 *Ph. chrysosporium*/SM-Tcn1 *S. moellendorffii*	682 aa	49.5	0.230 (HT)

PpatensLTR1 *P. patens*/SM-Tcn1 *S. moellendorffii*	675 aa	58.6	0.429

Pyggy			

PyrTriTy3-2 *Pyrenophora tritici-repentis*/NecHaemTy3-4*Nectria haematococca*	706 aa	77.0	0.259 (HT)

PyrTriTy3-2 *P. tritici-repentis*/ChaGloTy3-8 *Chaetomium globosum*	706 aa	48.5	0.706

PyrTriTy3-2*P. tritici-repentis*/grh *Magnaporthe grisea*	701 aa	49.2	0.703

PyrTriTy3-2 *P. tritici-repentis*/Dane4*Aspergillus nidulans*	695 aa	55.5	0.531

AltBraTy3-2*Alternaria brassicicola*/NecHaemTy3-4*N. haematococca*	709 aa	47.7	0.880

AltBraTy3-2*A. brassicicola*/Dane4 *A. nidulans*	695 aa	49.2	0.649

grh *M. grisea*/Dane4 *A. nidulans*	695 aa	48.5	0.670

Pyret			

skippy *Fusarium oxysporum*/PyrTriTy3-1 *P. tritici-repentis*	648 aa	40.4	0.772

skippy *F. oxysporum*/AFLAV *Aaspergillus flavus*	673 aa	41.4	0.766

AFLAV *A. flavus*/PyrTriTy3-1 *P. tritici-repentis*	648 aa	44.3	0.656

^a ^The corresponding accession numbers in GenBank are provided in the brackets;

^b ^ND - not determined: information concerning time divergence between species groups is unavailable; HT - putative horizontal transmission

It seems to be that the hypothesis of 'retained' genes cannot be implemented as explanation for Tcn1 clade distribution since investigated RT-Int fragments of Tcn1-like LTR retrotransposons from fungi and plants have higher similarity to each other than functional proteins which were proposed to be 'retained' [28]. RT-Int fragments from Tcn1-like LTR retrotransposons have average similarity 49%. Moreover, evolutionary rates estimated for Tcn1 LTR retrotransposons appeared to be less than evolutionary rates for 'retained' genes or other LTR retrotransposons (Table 3).

## Discussion

Despite a number of whole genome sequence studies, the distribution and diversity of CHD-containing Gypsy LTR retrotransposons is still poorly understood. Current knowledge of distribution and evolution of this group of mobile elements has been mainly obtained from diverse model organisms [3]. The quickly generated massive data sets (for example, WGS and EST databases) provide a great opportunity to perform detailed analysis for non-model organisms. Experimental data accumulation also should not be neglected. In the absence of information about genomic sequences from monilophytes, PCR screening seems to be very useful for isolation and characterization of new LTR retrotransposons from this group of non-seed vascular plants. Two new well-supported clades, Galahad and Mordred, as well as several other previously unknown lineages of CHD-containing Gypsy LTR retrotransposons were described based on the results of PCR-mediated survey of RT fragments from ferns, horsetails and lycophytes.

**Figure 5:**
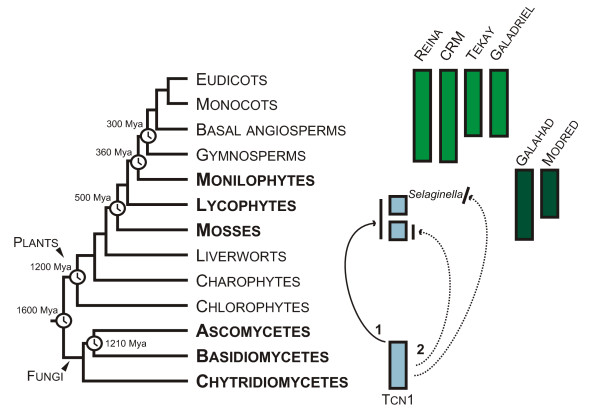
**Distribution of different clades of CHD-containing Gypsy LTR retrotransposons in plants**. Evolutionary tree is represented according to Bowman et al., 2007 [40] and Berbee and Taylor, 2001 [41] with minor modifications. Divergence times (Mya - million years ago) are indicated according to Hedges, 2002 [27]. Data suggest that Tcn1-like LTR retrotransposons were horizontally transmitted between fungi and non-seed plants (indicated by arrows). Presumably HT took place among fungi and the last common ancestor (LCA) of mosses and lycophytes (indicated as 1). Alternatively, it is possible that two independent acts of HT occurred (indicated as 2). First HT event could happen among fungi and LCA of mosses since all investigated mosses contain Tcn1-like LTR retrotransposons. The second HT could occur among fungi and LCA of *Selaginella *since only representatives of this genus carry this group of retrotransposons among all the investigated lycophytes.

One of the clades originally described for fungal genomes, Tcn1, appeared to be present in genomes of mosses (Bryophytes) and lycophytes (genus *Selaginella*) (Figure 5). Such an interkingdom distribution is not typical for CHD-containing LTR retrotransposons clades which are usually very specific for a particular taxonomic group [see [8]]. Data suggested that horizontal transmission took place between fungi and non-seed plants (probably mosses and lycophytes). Horizontal transmissions or horizontal transfers (HTs) of mobile elements are usually recognized by the presence of very closely related mobile elements in distant host taxa [29-33]. HT is well known for gypsy LTR retrotransposons in *Drosophila *[30] and has been suggested to have occurred in plants [2,24]. Recently, the evidence was provided for HT of RIRE1 LTR retrotransposon between representatives of genus *Oryza *[32] and Route66 LTR retrotransposon between representatives of Panicoideae (Poaceae) and several species of the genus *Oryza *[33].

Several criteria can be used for HT event recognition. The first criterion is inconsistencies between the phylogenies of transposable elements (TEs) and host species [29,34]. There are potential problems with application of this criterion for HT detection. Multiple transposable element lineages can be present within genomes. Moreover, transposable elements are multicopy components of genomes. Comparisons of paralogous copies instead of orthologs along with varying rates of their sequence evolution are the main sources for incongruence in phylogenetic analysis, this could be misidentified as HT. The second criterion, which seems to offer the strongest evidence, is a higher degree of observed sequence similarity for transposable elements than for functional genes, so called 'slowdown effect on evolutionary rates'. Once inserted, a new copy of transposable element is presumed to evolve without functional constrains. Thus, all types of mutations should have an equal chance to be fixed [35]. The lower than expected sequence divergence of TEs in comparison with non-mobile nuclear genes of the host species can be explained either by strong selective constraints in TE sequence coupled with a strict vertical transmission, or by horizontal transfer [31,36,37]. The third criterion of inferring HT is the discontinuous distribution of TEs among closely related taxa, i.e., presence of a TE in one lineage and its absence in a sister lineage. Such discontinuous distribution could be due to random loss of TEs, ancestral polymorphism, or independent sorting of copies into descendant species. By itself, this kind of evidence provides only weak support for HT since TE can be lost through population dynamics or ecological forces that are difficult to reconstruct [38,39].

All three criteria are satisfied in case of Tcn1-like LTR retrotransposons. They demonstrated patchy distribution among fungi and plants (Figure 5). They were found in all investigated mosses, but only in a few lycophytes and they absent in basal lineages of green plants such as green and red algae as well as in all seed plants investigated so far. The hypothesis of 'retained' genes in moss *Physcomitrella *represented an attractive alternative to horizontal transmission as an explanation of the phylogenetic inconsistencies as well as the existence of a number of functional genes in *Physcomitrella *genome, which seem to have non-plant origin and can be found in bacteria, fungi and protozoa but not in higher plants [28]. Nevertheless, the most important feature of Tcn1-like LTR retrotransposons in the context of HT is their lower evolutionary rates in comparison with other groups of CHD-containing LTR retrotransposons. The close examination and comparison of evolutionary rates for LTR retrotransposons including representatives of Tcn1, Pyggy and Pyret clades, and evolutionary rates estimated for putatively 'retained' genes suggests that a horizontal transmission of Tcn1-like LTR retrotransposons took place among fungi and the last common ancestor (LCA) of mosses and lycophytes (Table 3 and Figure 5) [27]. Alternatively, it is possible, but highly unlikely, that two independent acts of HT occurred. First HT event could happen among fungi and LCA of mosses since all investigated mosses contain Tcn1-like LTR retrotransposons [8]. The second HT could occur among fungi and LCA of *Selaginella *since only representatives of this genus carry this group of retrotransposons among all investigated lycophytes (Figure 4 and Figure 5). It is necessary to note that despite HT seeming to be a preferable explanation for the observed distribution. The evidence is not strong enough to discard other explanations; such as selective pressure coupled with vertical transmission of retrotransposons in genomes of non-seed plants and loss of these elements by other plants.

Another putative case of HT based on the results of present survey of LTR retrotransposons from fungal species was found for PyrTriTy3-2 LTR retrotransposon from *Pyrenophora tritici-repentis *Pt-1C-BFP (Dothideomycetes). PyrTriTy3-2 belongs to Pyggy clade and appeared to be more closely related to LTR retrotransposons from Sordariomycetes (NecHaemTy3-4 from *Nectria haematococca *MPVI and ChaGloTy3-8 from *Chaetomium globosum *CBS 148.51) than to the elements from other Dothideomycetes such as AltBraTy3-2 from *Alternaria brassicicola *ATCC 96866, REAL from *Alternaria alternata *(AB025309) [42], and PYGGY from *Pyrenophora graminea *(AF533704) [43] (Figure 2). The pairwise comparisons of RT-Int fragments and investigation of evolutionary rates for retrotransposons from Pyggy and Pyret clades revealed the unexpectedly high similarity between PyrTriTy3-2 and NecHaemTy3-4 (77% identical amino acids), much higher than between any other retrotransposons from Pyggy or Pyret clades, and at least two times lower evolutionary rate in the couple PyrTriTy3-2/NecHaemTy3-4 than in comparisons of other LTR retrotransposons (Table 3).

The high similarity, phylogenetic inconsistencies, as well as lower evolutionary rates could be explained by very strict evolutionary constraints or a HT event. However, taking into consideration that the high selective pressure could be implemented only in the case of functional importance of the PyrTriTy3-2 or NecHaemTy3-4, HT looks more preferable for the explanation of the described case. It is known that transposable elements can alter gene expression since they carry their own regulatory sequences and insertions can be selectively advantageous. However, only those transposable elements, which were involved in regulation, evolve under strict selective pressure [44,45].

While extremely rare, horizontal transfer seems to be quite common and recurrent in eukaryotes. An incomplete list of putative HT events includes: HT as a key event in the evolution of several fungal genes [46-48]; HT from fungi to rice weevil *Sitophilus oryzae *proposed for pectinase gene [49]; numerous HT events described for eukaryotic transposable elements [30-38]; as well as HTs of mitochondrial genes, for example, multiple angiosperm-angiosperm HTs of homing group I intron in the mitochondrial *cox1 *gene (for a review, see [50]); and a HT of the intron II and two adjacent exons of the mitochondrial *nad1 *gene from the flowering plants (angiosperms) to *Gnetum *(gymnosperms) [51].

The actual mechanisms of horizontal transfer for eukaryotic genes and transposable elements are still unknown since it is not possible to show experimentally how HT can occur. Parasites, symbionts, bacteria, or viruses all could be suggested as potential vectors for horizontal transfer. Moreover, based on an example of massive HT from a land plant donor to the basal angiosperm *Amborella trichopoda*, it has been demonstrated that direct plant-to-plant transfer can take a place [52]. The associations between biotrophic fungi and their plant hosts are ubiquitous in nature and range from mutually beneficial to potentially fatal pathogenic interactions. Mycorrhiza refers to an association or symbiosis between plants and fungi that colonize the cortical tissue of plant roots. Ectomycorrhizal fungi are mostly basidiomycetes that grow between root cortical cells of many tree species whereas arbuscular mycorrhizal (AM) fungi belong to the order Glomales (Glomeromycota) and form highly branched structures called arbuscules, within root cortical cells of wide range of land plant species [53-55]. Both types of mycorrhiza represent intimate association and could provide suitable conditions for HT of transposable elements. AM-like mycorrhiza is widely distributed among mosses, ferns and lycophytes (for review, [53]).

## Conclusions

Tcn1-like LTR retrotransposons were found in basidiomycota fungi and non-seed plants, including all investigated mosses and lycophytes from genus *Selaginella*. Such interkingdom distribution is not typical for chromodomain-containing LTR retrotransposons clades which are usually very specific for a particular taxonomic group and can be explained by strong selective constraints and the 'retained' genes theory or by horizontal transmission. The close examination and comparison of evolutionary rates for LTR retrotransposons including representatives of Tcn1 and two other clades of LTR retrotransposons, and evolutionary rates estimated for putatively 'retained' genes from mosses and fungi suggests that a horizontal transmission of Tcn1-like LTR retrotransposons took place among fungi and mosses/lycophytes. However the evidence is not strong enough to discard other explanations; such as selective pressure coupled with vertical transmission of retrotransposons in genomes of non-seed plants and loss of these elements by other plants.

## Methods

### Genomic sequences screening, sequence and phylogenetic analysis

Fungal genomic sequences are available at: Fungal Genome Initiative [56]; The DOE Joint Genome Institute [57]; and The Sanger Institute [58]. The source of individual genomes can be found in table represented in Additional file 5.

We used UniPro uGENE software [59] for LTR retrotransposons identification. The designed pipeline for Gypsy LTR retrotransposons identification and classification included: loading genomic sequence, translation of genomic sequence over six possible reading frames to amino acids, and subsequent search for homologous regions performed using "HMMER search" options of UniPro uGENE. The algorithm of HMMER search is based on profile hidden Markov models, which can perform amino acid sequence searches by use of an appropriate profile [60]. For the analyses, we used a multiple alignment consensus sequence, which contains Gypsy LTR retrotransposon reverse transcriptase (RT) and partial integrase (Int) domains. The profile HMM, based on this consensus sequence, was built using UniPro uGENE software. An additional test for the presence of RT and partial Int domains was performed using BLAST (blastp) which also was incorporated in the designed pipeline. All BLAST analysis was essentially performed using sequence databases accessible from the National Center for Biotechnology Information [61]. The classification of the newly identified elements was performed by a comparative analysis of their sequences. Newly identified elements and their accession numbers in public databases are listed in Additional file 6.

The whole nucleotide sequences of the transposable elements, if possible, were also extracted with the assistance of UniPro uGENE software. After localization of amino acid sequences obtained during HMMER search in the initial genomes in its nucleotide representation, the sequences were expanded up to 15 Kb and used for long terminal repeats (LTRs) search. The algorithm for repeats search, 'Repeat Find', is included to the UniPro uGENE as well as the visualization feature and 'ORF Find' option which were used to identify the putatively intact copies of LTR retrotransposons. Structural features of newly identified LTR retrotransposons can be found in Additional file 1.

Tcn1-like LTR retrotransposon search was carried out using BLAST (blastp and blastx). BLAST analysis was performed using sequence databases accessible from the National Center for Biotechnology Information (NCBI) server [59], The U.S. Department of Energy Joint Genome Institute [57], and Broad Institute of MIT and Harvard [56] as well as Phytozome, a tool for green plant comparative genomics [62]. The described copy of SM-Tcn1 from spikemoss *Selaginella moellendorffii *(Lycopodiophyta) is located in scaffold_0 (1426925-1421008) of genomic sequence version 1.0 which is available at The U.S. Department of Energy Joint Genome Institute web-site [57]. The whole sequence of SM-Tcn1 with annotations can be found in Additional file 3. Other websites used in the present study were: Repbase [63], NCBI conserved domain database and search service [64], ESTs from *Porphyra yezoensis *at Kazusa DNA Research Institute [65], *Cyanidioschyzon merolae *Genome Project [66], The Plant Genomics Consortium [67], The Institute for Genomic Research [68], Cassava and Leafy Spurge EST Project [69].

All multiple DNA alignments were performed by ClustalW [70] and edited manually in UniPro uGENE. Phylogenetic analyses were performed using the Neighbor-Joining (NJ) method in MEGA 4.0 program [71]. Statistical support for the NJ tree was evaluated by bootstrapping (number of replications, 1000) [72]. Evolutionary rates were estimated by standard methods [73]. Poisson correction distances (d) were estimated from the equation d = -ln(1 - p), where p represents the proportion of different amino acids. The rate of amino acid substitution (r) was estimated by the standard equation r = d/2T, where T is the divergence time of the last common ancestor of the compared species. The estimated divergence times used were: Plants/Fungi, 1500 Myr and Basidiomycetes/Ascomycetes, 1200 Myr according to Hedges (2002) [27]; Homobasidiomycetes/Chytridiomycetes, 900 Myr, Sordaryomycetes/Eurotiomycetes, 540 Myr, and Sordaryomycetes/Dothideomycetes, 490 Myr according to Padovan et al. (2005) [74]; and Heterobasidiomycetes (or Tremellomycetes)/Homobasidiomycetes (Agaricomycetes and Dacrymycetes), 700 Myr according to Hibbett et al. (2007) [75] and Taylor et al. (2004) [76].

### Species collection and total DNA isolation

Table 2 lists plant species, and Table 3 lists fungal species used in present study. The taxonomy of vascular non-seed plants (monilophytes and lycophytes) is given after Pryer et al. (2004) [22], Smith et al. (2006) [21], and Korall et al. (2007) [77]. Plant species (monilophytes) were collected in nature. The detailed label data are available from the authors. The genomic DNA of lycophytes was provided by the Royal Botanic Gardens, Kew, London, UK [78]. Genomic DNA was isolated from the leaves. Extraction was performed using the QIAGEN DNeasy Plant Mini Kit (QIAGEN). Isolated DNA was used directly in PCR amplifications.

### Gypsy LTR retrotransposons PCR amplification and sequencing

Previously designed degenerate PCR primers for chromodomain-containing Gypsy LTR retrotransposons were used in present study: GyRT1 = 5'-MRNATGTGYGTNGAYTAYMG-3' [24] and ty3-A = 5'-AATTCGCTGCCGCTAAGATNARNADRTCRTC-3' [8], where M = A + C, Y = C + T, R = A + G, D = A + G + T and N = A + G + C + T. These primers were designed to amplify the most conserved part of the reverse transcriptase (RT) domain of LTR retrotransposons and were proved to be efficient [8,24]. The expected length of PCR products was about 320 bp. PCR amplification with degenerate primers was performed using 0.1 μg of genomic DNA in 10-μl volume of 10 mM Tris-HCl (pH 8.9), 1 mM (NH_4_)_2_SO_4_, 4 mM MgCl_2_, 200 μM each of four dNTPs, 0.5 μM primers, and 2.5 units of Taq polymerase. After an initial denaturation step for 3 min at 94°C, the PCR reactions were subjected to 30 cycles of amplification consisting of 30 sec denaturation at 94°C, 42 sec annealing at 50°C, and 1 min extension at 72°C. PCR products were separated by agarose gel electrophoresis. The resulting PCR products were directly ligated into a pGEM vector using a pGEM-T-Easy cloning kit (Promega) for sequence determination.

Clones were amplified by PCR with M13 primers, and 40 ng of the product was used in a 10 μl cycle sequencing reaction with the ABI BigDye Terminator Kit on an ABI 310 Genetic Analyser (Applied Biosystems); or sequencing reactions were performed with Dye Terminator Cycle Sequencing Kit (Beckman Coulter) and analyzed on CEQ 8000 Genetic Analysis System. Sequences were deposited to GenBank under Acc. Numbers GQ443314-GQ443445 and AY959294-AY959313.

## List of abbreviations used

LTR: long terminal repeat; SINE: short interspersed nuclear element; CHD: chromodomain; RT: reverse transcriptase; Int: integrase; ORF: open reading frame; PR: proteinase; dUTPase: deoxyuridine triphosphatase domain; PPT: polypurine tract; PBS: primer-binding site; HT: horizontal transfer or horizontal transmission; TE: transposable element; LCA: last common ancestor; AM: arbuscular mycorrhiza; HMM: hidden Markov models; Myr: million years; Mya: million years ago.

## Authors' contributions

ON participated in the design of the study, carried out the analysis, participated in the sequence analysis and drafted the manuscript. GS contributed to data acquisition, participated in analysis and interpretation. AB participated in the design of the study, performed coordination and has given final approval of the submitted version. All authors read and approved the final manuscript.

## Supplementary Material

Additional file 1 **Structure of novel LTR retrotransposons from Fungi**. Table contained list of novel LTR retrotransposons from Fungi detected in present study, their copy numbers and putative structure including predicted enzymatic domains. Click here for file

Additional file 2 **Phylogenetic analysis of dUTPase**. Neighbor-Joining phylogenetic tree reconstructed based on dUTPase amino acid sequences from eukaryotes, viruses, and dUTPase domains from CHD-containing Gypsy LTR retrotransposons. Click here for file

Additional file 3 **SM-Tcn1 CHD-containing Gypsy LTR retrotransposon**. Sequence of SM-Tcn1 CHD-containing Gypsy LTR retrotransposon from spikemoss *Selaginella moellendorffii *(Lycopodiophyta) with annotations in GenBank format. Click here for file

Additional file 4 **Phylogenetic analysis of Athila-like LTR retrotransposons**. Neighbor-joining (NJ) phylogenetic tree based on RT nucleotide sequences of Athila-like LTR retrotransposons including newly described elements. Click here for file

Additional file 5 **List of fungal species, genomes of which were analyzed**. Table contained the list of fungal species, genomes of which were analyzed *in silico *in the present study and the sources of genomic sequences. Click here for file

Additional file 6 **Novel Gypsy LTR retrotransposons from Fungi**. Table contained the list of novel Gypsy LTR retrotransposons from Fungi detected in present study and their accession numbers. Click here for file
