# Supplementary material for: Evolutionary genomics revealed interkingdom distribution of Tcn1-like chromodomain-containing Gypsy LTR retrotransposons among fungi and plants
Source: BMC Genomics. 2010 Apr 8;11:231. doi: 10.1186/1471-2164-11-231 (PMC2864245; doi:10.1186/1471-2164-11-231)
Supplement: Additional file 1 — Structure of novel LTR retrotransposons from Fungi. Table contained list of novel LTR retrotransposons from Fungi detected in present study, their copy numbers and putative structure including predicted enzymatic domains. [file 1471-2164-11-231-S1.DOC]

**Additional Table S1.** Novel Gypsy LTR retrotransposons from Fungi detected in present study, their copy number and putative structure including predicted enzymatic domains (for the intact element or retrotransposons carried long coding regions)

| Sp.^1^ | type^2^/clade | element  (copies^3^) | IE^4^ | length  (bp) | LTRs  length / homology | TSD (length) | Putative solo LTRs (number) | Gag domains^6^ | Pol domains^6^ | Additional ORFs (if present) |
| --- | --- | --- | --- | --- | --- | --- | --- | --- | --- | --- |
| Cg | Chr/Maggy | **ChaGloTy3-1** (4) | + | 6744 | 179 bp / 100% | + (5 bp) | + (2) | Retrotrans_gag-CCHC | RVP_2-RT-RNH-Int(GPY/F)-chromo |  |
|  | Chr/Maggy | ChaGloTy3-2 (1) | -- | 6652 | 196 bp / 100% | + (5 bp) | -- | -- | RT-RNH-Int(GPY/F)-chromo |  |
|  | Chr/Maggy | **ChaGloTy3-3** (5) | + | 6642 | 196 bp / 100% | + (5 bp) | + (16) | Retrotrans_gag-CCHC | RT-RNH-Int(GPY/F)-chromo |  |
|  | Chr/Pyret | ChaGloTy3-4 (6) | -- | 7448 | 212 bp / 93.9% | -- | + (>180) | Retrotrans_gag-CCHC | RT-Int(HHCC,GPY/F)-chromo |  |
|  | Chr/Afut4 | **ChaGloTy3-5a** (1)  **ChaGloTy3-5b** (2) | +  +?^5^ | 7549  7466^5^ | 223 bp / 100%  171 bp / 100% | + (5 bp)  + (5 bp) | + (7)  + (10) | CCHH-Herpes_UL51[COG0127] ^7^-RT-Int(GPY/F)-chromo  CCHH-Herpes_UL51[COG0127] ^7^-RT-Int(GPY/F)-chromo | |  |
|  | Chr/Afut4 | **ChaGloTy3-6** (1) | +?^5^ | 7193^5^ | 106 bp / 94.3% | -- | + (2) |  | RT-Int(GPY/F)-chromo |  |
|  | Chr/Yeti | **ChaGloTy3-7** (1) | + | 7134 | 300-306 bp / 90.9% | -- | + (12) |  | RT-Int(HHCC,GPY/F) |  |
|  | Chr/Pyggy | ChaGloTy3-8 (1) | -- | ND | ND | -- | -- | Retrotrans_gag | RT-Int(HHCC)-chromo |  |
| Fo | Chr/Maggy | **FusOxyTy3-1** (15) | + | 5685 | 238 bp / 100% | + (5 bp) | + (6) | CCHC | RVP_2-RT-Int(HHCC,GPY/F)-chromo |  |
|  | Chr/Maggy | **FusOxyTy3-2** (8) | + | 5739 | 242 bp / 100% | + (5 bp) | + (8) | Retrotrans_gag-CCHC | RVP_2-RT-Int(HHCC,GPY/F)-chromo |  |
|  | Chr/Afut1 | FusOxyTy3-3 (6) | -- | 9598 | 915-916 bp / 95.3% | -- | + (>90) |  | RT-Int |  |
| Fv | Chr/Afut1 | FusVerTy3-1 (2) | -- | ND^5^ | ND | -- | -- |  | RT-Int |  |
|  | Chr/Afut1 | FusVerTy3-2 (2) | -- | ND^5^ | ND | -- | -- |  | RT-Int |  |
| Nh | Chr/Afut1 | NecHaemTy3-1 (45) | -- | 7993 | 386 bp / 90.9% | -- | -- |  | RT-Int |  |
|  | Chr/Afut4 | NecHaemTy3-2 (8) | -- | 10068 | 237 bp / 99.2% | + (5 bp) | + (3) | -- | RT-Int(GPY/F)-chromo |  |
|  | Chr/Nessie | NecHaemTy3-3 (6) | -- | 7665 | 202 bp / 99.5% | + (5 bp) | -- | Retrotrans_gag | RT-Int-chromo |  |
|  | Chr/Maggy | **NecHaemTy3-4** (2) | + | 5349 | 169 bp / 100% | -- | + (2) |  | RT-Int-chromo |  |
|  | Ylt1 | **NecHaemTy3-5** (2) | + | 12164 | 312-313 bp /99.7% | -- | + (9) | RVP_2 | RT-RNH-Int(HHCC,GPY/F) | add. ORF - unknown |
|  | Ylt1 | NecHaemTy3-6 (1) | -- | ND^5^ | ND | -- | -- |  | RT-Int |  |
| Pa | Chr/Afut1 | PodAnsTy3-1 (8) | -- | ND^5^ | ND | -- | -- |  | RT-Int |  |
|  | Chr/Yeti | **PodAnsTy3-2** (2) | + | 6937 | 355-353 bp / 99.4% | + (5 bp) | + (5) | Retrotrans_gag-RT-Int(HHCC,GPY/F)-chromo | |  |
|  | Chr/Mgrl3 | PodAnsTy3-3 (1) | -- | ND | ND | -- | -- |  | RT-Int(HHCC) |  |
| Tr | Chr/Polly | TrichReeTy3-1 (2) | -- | ND^5^ | ND | -- | -- |  | RT-Int |  |
|  | Chr/Afut1 | TrichReeTy3-2 (1) | -- | ND | ND | -- | -- |  | RT-Int |  |
|  | Chr/Afut4 | TrichReeTy3-3 (1) | -- | ND | ND | -- | -- |  | RT-Int |  |
|  | Chr/Afut4 | TrichReeTy3-4 (1) | -- | 8768 | 157 bp / 90.4% | + (5 bp) | -- |  | RT-Int |  |
| Tv | Chr/Afut1 | TrichVirTy3-1(5) | -- | 7327 | 411 bp / 85.2% | + (5 bp) | + (1) |  | RT-Int |  |
| Ac | Chr/Afut1 | AspClaTy3-1 (23) | -- | 6877 | 257-258 bp / 89.5% | + (5 bp) | + (>20) |  | RT-Int |  |
|  | Chr/Afut1 | AspClaTy3-2 (2) | -- | ND^5^ | ND | -- | -- |  | RT-Int |  |
|  | Chr/Afut1 | AspClaTy3-3 (1) | -- | ND | ND | -- | -- |  | RT-Int |  |
|  | Chr/Afut4 | AspClaTy3-4 (2) | -- | ND^5^ | ND | -- | -- |  | RT-Int |  |
|  | Chr/Afut4 | AspClaTy3-5 (1) | -- | ND | ND | -- | -- |  | RT-Int |  |

**Additional Table S1.** **(continued).** Novel Gypsy LTR retrotransposons from Fungi detected in present study, their copy number and putative structure including predicted enzymatic domains (for the intact element or retrotransposons carried long coding regions)

| Sp.^1^ | type^2^/clade | element  (copies^3^) | IE^4^ | length  (bp) | LTRs  length / homology | TSD (length) | Putative solo LTRs (number) | Gag domains^6^ | Pol domains^6^ | Additional ORFs (if present) |
| --- | --- | --- | --- | --- | --- | --- | --- | --- | --- | --- |
| Ani | Chr/Afut4 | AspNigTy3-1 (1) | -- | 6670 | 160 bp / 94.4% | + (5 bp) | -- |  | RT-Int |  |
| At | Chr/Afut1 | AspTerTy3-1 (3) | -- | 7752 | 678 bp / 87.2% | + (5 bp) | -- |  | RT-Int |  |
| Ci | Chr/Afut1 | CocImmTy3-1 (77) | -- | 7067 | 296 bp / 75.0% | + (4 bp) | + (>50) |  | RT-RNH-Int |  |
|  | Chr/Afut1 | CocImmTy3-2 (40) | -- | 6956 | 263 bp / 87.5% | + (5 bp) | + (>50) |  | RT-Int(HHCC)-chromo |  |
|  | Chr/Coccy1 | CocImmTy3-3 (39) | -- | 7661 | 577 bp / 92.9% | + (3 bp) | -- |  | RT-Int |  |
|  | Chr/Afut1 | CocImmTy3-4 (3) | -- | ND^5^ | ND | -- | -- |  | RT-Int |  |
|  | Chr/Coccy2 | CocImmTy3-5 (2) | -- | ND^5^ | ND | -- | -- |  | RT-Int |  |
|  | Chr/Polly | CocImmTy3-6 (11) | -- | 5359 | 214 bp / 86.4% | -- | -- |  | RT-Int |  |
|  | Chr/Afut4 | CocImmTy3-7 (2) | -- | ND^5^ | ND | -- | -- |  | RT-Int |  |
|  | Ylt1 | CocImmTy3-8 (64) | -- | 9044 | 430 bp / 92.6% | + (5 bp) | -- | CCHC-RVP_2 | RT-Int |  |
| Hc | Chr/Nessie | **HisCapTy3-1** (13) | + | 7532 | 556 bp / 99.5% | + (5 bp) | + (>50) | CCHC | RVP_2-RT-Int(HHCC,GPY/F)-chromo |  |
|  | Chr/Nessie | **HisCapTy3-2** (4) | + | 6226 | 435 bp / 100% | + (5 bp) | + (>30) |  | RVP_2-RT-Int(HHCC,GPY/F)-chromo |  |
|  | Chr/Polly | HisCapTy3-3 (11) | -- | 4480 | 350-366 bp / 74.7% | -- | + (12) |  | RT-Int |  |
|  | Chr/Nessie | **HisCapTy3-4** (9) | + | 6709 | 289 bp / 98.3% | -- | + (>50) |  | RT-Int(HHCC,GPY/F)-chromo |  |
|  | Chr/Nessie | **HisCapTy3-5** (6) | + | 7445 | 554 bp / 100% | + (5 bp) | -- | CCHC | RVP_2-RT-Int(HHCC,GPY/F)-chromo |  |
|  | Chr/Nessie | **HisCapTy3-6** (24) | + | 6565 | 216 bp / 100% | -- | + (>40) | CCHC | RT-Int(HHCC,GPY/F)-chromo |  |
|  | Chr/Nessie | HisCapTy3-7 (1) | -- | ND | ND | -- | -- |  | RT-Int |  |
|  | Chr/Afut4 | HisCapTy3-8 (1) | -- | ND^5^ | 169 bp / 94.7% | + (5 bp) | -- |  | RT-Int |  |
|  | Chr/Afut4 | HisCapTy3-9 (1) | -- | ND | ND | -- | -- |  | RT-Int |  |
|  | Chr/Afut4 | **HisCapTy3-10** (2) | + | 8596 | 238 bp / 99.2% | + (5 bp) | -- |  | RT-Int(GPY/F)-chromo |  |
|  | Chr/Afut4 | HisCapTy3-11 (1) | -- | ND | ND | -- | -- |  | RT-Int |  |
| Ur | Chr/Coccy2 | UncReeTy3-1 (13) | -- | 5771 | 227-228 bp / 89% | + (5 bp) | + (5) |  | RT-Int |  |
|  | Chr/Polly | UncReeTy3-2 (9) | -- | ~5840 | 284 bp / 87.3% | + (5 bp) | + (10) |  | RT-Int |  |
|  | Chr/Afut1 | UncReeTy3-3 (6) | -- | ND | ND | -- | -- |  | RT-Int |  |
|  | Chr/Coccy1 | UncReeTy3-4 (20) | -- | 7579 | 593 bp / 85.2% | -- | -- |  | RT-Int |  |
|  | Chr/Nessie | UncReeTy3-5 (9) | -- | 7610 | 252-253 bp / 84.6% | -- | -- |  | RT-Int |  |
|  | Chr/Nessie | UncReeTy3-6 (7) | -- | 6945 | 348 bp / 86.5% | + (3 bp) | -- |  | RT-Int |  |
| Ss | Chr/Pyret | **ScleSclerTy3-1** (10) | + | 6112 | 117 bp / 94.9% | -- | + (>100) | CCHC-RVP_2 | RT-Int(HHCC,GPY/F)-chromo |  |
|  | Chr/Pyret | **ScleSclerTy3-2** (3) | + | 5700 | 403 bp / 100% | + (5 bp) | + (>30) | Retrotrans_gag-CCHC-RVP_2 | RT-Int(GPY/F)-chromo |  |
|  | Chr/Mgrl3 | **ScleSclerTy3-3** (6) | + | 5621 | 148 bp / 100% | + (5 bp) | + (>150) | Retrotrans_gag-CCHC | RVP_2-RT-Int(HHCC,GPY/F)-chromo |  |
|  | Chr/Mgrl3 | **ScleSclerTy3-4** (6) | + | 6573 | 328 bp / 99.1% | + (5 bp) | -- | -- | RVP_2-RT-Int(HHCC,GPY/F)-chromo |  |

**Additional Table S1.** **(continued).** Novel Gypsy LTR retrotransposons from Fungi detected in present study, their copy number and putative structure including predicted enzymatic domains (for the intact element or retrotransposons carried long coding regions)

| Sp.^1^ | type^2^/clade | element  (copies^3^) | IE^4^ | length  (bp) | LTRs  length / homology | TSD (length) | Putative solo LTRs (number) | Gag domains^6^ | Pol domains^6^ | Additional ORFs (if present) |
| --- | --- | --- | --- | --- | --- | --- | --- | --- | --- | --- |
| Bc | Chr/Pyret | **BotCinTy3-1** (17) = Boty | + | 6604 | 561-570 bp / 93.9% | + (5 bp) | + (8) | Retrotrans_gag-CCHC-RVP_2-RT-Int(HHCC,GPY/F)-chromo | |  |
|  | Chr/Nessie | **BotCinTy3-2** (2) | + | 7380 | 165 bp / 100% | -- | -- | Retrotrans_gag-CCHC | RT-Int(HHCC,GPY/F)-chromo |  |
|  | Chr/Maggy | **BotCinTy3-3** (4) | + | 6439 | 149 bp / 100% | + (5 bp) | + (4) | CCHC | RVP_2-RT-RNH-Int(GPY/F)-chromo |  |
|  | Ylt1 | **BotCinTy3-4** (2) | + | 10972 | 273 bp / 100% | -- | -- | RVP_2 | RT-Int(HHCC,GPY/F) | add. ORF - partial RT [cd01644] ^7^ |
| Abr | Chr/Polly | AltBraTy3-1 (6) | -- | 6707 | 242 bp / 92.6% | + (3 bp) | + (>30) |  | RT-Int |  |
|  | Chr/Pyggy | AltBraTy3-2 (8) | -- | 7247 | 481 bp / 95.0% | + (5 bp) | + (3) |  | RT-Int |  |
|  | Chr/Polly | AltBraTy3-3 (3) | -- | ND^5^ | ND | -- | -- |  | RT-Int |  |
|  | Chr/Polly | AltBraTy3-4 (11) | -- | ND^5^ | ND | -- | -- |  | RT-Int |  |
|  | Chr/Afut1 | AltBraTy3-5 (19) | -- | 7111 | 404 bp / 95.0% | + (4 bp) | -- |  | RT-Int |  |
|  | Ylt1 | AltBraTy3-6 (13) | -- | 13357 | 219-223 bp / 90.1% | + (3 bp) | + (>30) |  | RT-Int |  |
| Ptr | Chr/Pyret | **PyrTriTy3-1** (11) | + | 6776 | 460 bp / 99.1% | -- | + (12) | CCHC | RVP_2-RT-RNH-Int(HHCC,GPY/F)-chromo |  |
| HT? | Chr/Pyggy | **PyrTriTy3-2** (39) | + | 5385 | 188 bp / 99.5% | + (5 bp) | + (13) |  | RT-Int-chromo |  |
|  | Chr/Maggy | **PyrTriTy3-3** (10) | + | 7018 | 176 bp / 100% | + (5 bp) | + (3) | RT-Int(HHCC,GPY/F)-chromo | |  |
|  | Chr/Maggy | **PyrTriTy3-4** (12) | + | 7228 | 208 bp / 100% | + (5 bp) | + (14) |  | RVP_2-RT-Int(HHCC,GPY/F)-chromo |  |
|  | Chr/Maggy | **PyrTriTy3-5** (14) | + | 7110 | 167 bp / 100% | + (5 bp) | + (>30) |  | RVP_2-RT-Int(HHCC,GPY/F)-chromo |  |
|  | Chr/Maggy | **PyrTriTy3-6** (1) | + | 8113 | 184 bp / 97.8% | -- | + (11) | CCHC | RVP_2-RT-Int(HHCC,GPY/F)-chromo |  |
|  | Chr/Maggy | **PyrTriTy3-7** (13) | + | 7873 | 179 bp / 100% | + (5 bp) | + (12) | CCHC | RVP_2-RT-Int(HHCC,GPY/F)-chromo |  |
|  | Ylt1 | **PyrTriTy3-8** (3) | + | 7970 | 212 bp / 100% | + (5 bp) | + (>30) | CCHC-RVP_2 | RT-Int(HHCC,GPY/F) |  |
| Sn | Chr/Afut1 | StaNodTy3-1 (5) | -- | 7361 | 200 bp / 90.5% | -- | + (15) |  | RT-Int |  |
|  | Chr/Afut1 | StaNodTy3-2 (2) | -- | 8965 | 315 bp / 94.9% | -- | + (19) |  | RT-Int |  |
|  | Chr/Polly | StaNodTy3-3 (3) | -- | ND^5^ | ND | -- | -- |  | RT-Int |  |
|  | Chr/Afut1 | StaNodTy3-4 (1) | -- | 7060 | 481 bp / 94.0% | -- | -- |  | RT-Int |  |
|  | Chr/Afut4 | StaNodTy3-5 (1) | -- | ND | 129 bp / 97.7% | -- | -- |  | RT-Int |  |
| Ab | Chr/MarY1 | AmaBisTy3-1 (7) | ?^5^ | ND^5^ | ND | -- | -- |  | RT-Int |  |
| Cc | Chr/Tcn1 | **CopCinTy3-1** (37) = **Ccchromovir-1** | + | 7837 | 829-827 bp / 98.8% | + (5 bp) | -- | Retrotrans_gag | RVP_2-RT-RNH-Int(HHCC)-chromo |  |
|  | Chr/MarY1 | CopCinTy3-2 (11) | -- | 7552 | 497-498 bp / 98.4% | + (5 bp) | + (10) | Retrotrans_gag-RVP_2-RT-Int(GPY/F)-chromo | |  |
|  | Chr/MarY1 | **CopCinTy3-3** (8) | + | 6743 | 449-450 bp / 94.9% | + (5 bp) | + (6) | Retrotrans_gag-CCHC | RVP_2-RT-Int(GPY/F)-chromo |  |
|  | Chr/MarY1 | CopCinTy3-4 (5) | ? ^5^ | 7058^5^ | 458-460 bp / 96.3% | + (5 bp) | + (7) |  | RVP_2-RT-Int(GPY/F)-chromo |  |
|  | Chr/MarY1 | CopCinTy3-5 (14) | -- | 6190 | 337 bp / 99.1% | + (5 bp) | + (5) | Retrotrans_gag-CCHC | RVP_2-RT-Int(GPY/F)-chromo |  |
|  | Chr/MarY1 | **CopCinTy3-6** (69) = **Ccchromovir-4** | + | 6903 | 539 bp / 100% | + (5 bp) | -- | Retrotrans_gag-CCHC | RVP_2-RT-RNH-Int(GPY/F)-chromo |  |
|  | Chr/MarY1 | CopCinTy3-7 (1) | -- | ND | ND | -- | -- | Retrotrans_gag-CCHC | RVP_2-RT-Int(GPY/F)-chromo |  |

**Additional Table S1.** **(continued).** Novel Gypsy LTR retrotransposons from Fungi detected in present study, their copy number and putative structure including predicted enzymatic domains (for the intact element or retrotransposons carried long coding regions)

| Sp.^1^ | type^2^/clade | element  (copies^3^) | IE^4^ | length  (bp) | LTRs  length / homology | TSD (length) | Putative solo LTRs (number) | Gag domains^6^ | Pol domains^6^ | Additional ORFs (if present) |
| --- | --- | --- | --- | --- | --- | --- | --- | --- | --- | --- |
| Cc | Chr/Tcn2 | **CopCinTy3-8** (4) = **Ccchromovir-5** | + | 5797 | 195 bp / 100% | + (5 bp) | -- | RVP_2-RT-RNH-Int(GPY/F)-chromo | |  |
|  | Chr/Tcn2 | CopCinTy3-9 (2) | -- | ND^5^ | ND | -- | -- | -- | RT-Int(GPY/F)-chromo |  |
|  | Chr/Tcn1 | **CopCinTy3-10** (2) = **Ccchromovir-2** | + | 8041 | 1104-1077 bp / 96.2% | + (5 bp) | + (7) | CCHC | RT-Int(HHCC,GPY/F)-chromo |  |
|  | Chr/Tcn2 | **CopCinTy3-11** (1) | + | 9496 | 269-268 bp / 99.6% | + (5 bp) | + (2) | Retrotrans_gag-CCHC-RVP_2-RT-Int(GPY/F) | |  |
|  | Chr/Tcn2 | CopCinTy3-12 (1) | -- | ND^5^ | ND | -- | -- |  | RT-Int |  |
|  | Chr/Laccy2 | **CopCinTy3-13** (1) | + | 12537 | 624 bp / 100% | + (5 bp) | + (1) |  | RT-Int(HHCC,GPY/F)-chromo |  |
|  | SN1006 | CopCinTy3-14 (2) | ? ^5^ | ND^5^ | ND | -- | -- |  | RT-Int |  |
|  | Ylt1 | CopCinTy3-15 (1) | -- | ND^5^ | ND | -- | -- |  | RT-RH-Int(GPY/F) |  |
|  | Ylt1 | CopCinTy3-16 (1) | -- | ND^5^ | ND | -- | -- |  | RT-Int |  |
| Lb | Chr/Laccy1 | **LacBicTy3-1** (4) | + | 5850 | 246 bp / 100% | -- | -- | Retrotrans_gag-CCHC | RT-Int(GPY/F)-chromo |  |
|  | Chr/MarY1 | LacBicTy3-2 (11) | -- | 7786 | 599 bp / 98.5% | + (5 bp) | + (8) | Retrotrans_gag-CCHC | RVP_2-RT-Int(GPY/F)-chromo | add. ORF - unknown |
|  | Chr/Tcn2 | **LacBicTy3-3** (5) | + | 5870 | 249 bp / 99.6% | + (5 bp) | -- | RVP_2 | RT-Int(GPY/F)-chromo |  |
|  | Chr/Tcn2 | **LacBicTy3-4** (4) | + | 8775 | 370 bp / 99.5% | -- | -- | Retrotrans_gag-CCHC | RVP_2-RT-Int(GPY/F) |  |
|  | Chr/Tcn2 | **LacBicTy3-5** (3) | + | 6479 | 169 bp / 99.4% | -- | -- |  | RVP_2-RT-Int(GPY/F) |  |
|  | Chr/Tcn2 | **LacBicTy3-6** (15) | + | 5840 | 234 bp / 100% | + (5 bp) | -- | RT-Int(GPY/F)-chromo | |  |
|  | Chr/Laccy2 | **LacBicTy3-7** (6) | + | 9823 | 694 bp / 100% | + (5 bp) | -- | CCHC | RT-Int(GPY/F)-chromo | add. ORF - unknown |
|  | Chr/Laccy2 | **LacBicTy3-8** (7) | + | 11988 | 582 bp / 99.8% | + (5 bp) | -- | RT-Int(HHCC,GPY/F)-chromo | | add. ORF - unknown |
|  | Chr/Laccy2 | **LacBicTy3-9** (4) | + | 12865 | 524 bp / 100% | + (5 bp) | -- | Birna_VP4[pfam01768] ^7^-RT-Int(HHCC) | | add. ORF - unknown |
|  | Chr/Laccy2 | **LacBicTy3-10** (6) | + | 11687 | 496 bp / 100% | + (5 bp) | -- | RT-Int(HHCC,GPY/F)-chromo | | add. ORF - unknown |
|  | Chr/Laccy2 | LacBicTy3-11 (3) | -- | 16187 | 544 bp / 97.6% | + (5 bp) | -- |  | RT-Int |  |
|  | Chr/Laccy2 | **LacBicTy3-12** (15) | + | 11186 | 383 bp / 100% | + (5 bp) | -- | Retrotrans_gag-CCHC | RT-Int(GPY/F)-chromo | add. ORF - unknown |
|  | Ylt1 | LacBicTy3-13 (10) | -- | ND^5^ | -- | -- | -- |  | RT-Int |  |
|  | Ylt1 | **LacBicTy3-14** (10) | + | 8807 | 768-770 bp / 97.9% | -- | -- |  | RT-Int(HHCC,GPY/F) |  |
|  | SN1006 | **LacBicTy3-15** (3) | + | 6655 | 147 bp / 100% | -- | -- |  | RT-Int |  |

**Additional Table S1.** **(continued).** Novel Gypsy LTR retrotransposons from Fungi detected in present study, their copy number and putative structure including predicted enzymatic domains (for the intact element or retrotransposons carried long coding regions)

| Sp.^1^ | type^2^/clade | element  (copies^3^) | IE^4^ | length  (bp) | LTRs  length / homology | TSD (length) | Putative solo LTRs (number) | Gag domains^6^ | Pol domains^6^ | Additional ORFs (if present) |
| --- | --- | --- | --- | --- | --- | --- | --- | --- | --- | --- |
| Pp | Chr/MarY1 | **PosPlaTy3-1** (>200) | + | 6823^5^ | 484 bp / 99.4% | + (5 bp) | -- |  | RVP_2-RT-Int(GPY/F)-chromo |  |
|  | Chr/MarY1 | **PosPlaTy3-2** (>100) | + | 7522^5^ | 837 bp / 91.3% | -- | -- | Retrotrans_gag-CCHC | RVP_2-RT-RNH-Int(GPY/F)-chromo |  |
|  | Chr/MarY1 | **PosPlaTy3-3** (>500) | + | 8810 | 398 bp / 98.2% | + (3 bp) | -- | Retrotrans_gag | dUTPase^7^-RVP_2-RT-Int(GPY/F)-chromo |  |
|  | Chr/MarY1 | **PosPlaTy3-4** (~40) | + | 7993 | 563 bp / 98.8% | + (3 bp) | -- | RVP_2-RT-Int(GPY/F)-chromo-dUTPase^7^ | |  |
|  | Chr/MarY1 | **PosPlaTy3-5** (>300) | + | 7241 | 399 bp / 100% | + (5 bp) | -- | CCHC-RVP_2-dUTPase^7^-RT-Int(GPY/F)-chromo | |  |
|  | Chr/Laccy1 | PosPlaTy3-6 (5) | -- | 8811 | 399 bp / 98.2% | -- | -- | Retrotrans_gag | RVP_2-RT-Int |  |
|  | Chr/Tcn2 | PosPlaTy3-7 (1) | ?^5^ | 5899^5^ | 254 bp / 97.6% | -- | -- |  | RT-Int |  |
|  | Chr/Tcn2 | PosPlaTy3-8 (1) | -- | ND | -- | -- | -- |  | RT-Int(GPY/F) |  |
|  | Ylt1 | PosPlaTy3-9 (>300) | ?^5^ | ND^5^ | -- | -- | -- |  | RT-Int(GPY/F) |  |
| Sr | Chr/Puccy2 | SpoRosTy3-1 (2) | ?^5^ | ND^5^ | -- | -- | -- |  | RVP_2-RT-Int(GPY/F)-chromo |  |
|  | Chr/Puccy2 | **SpoRosTy3-2** (2) | + | 8177 | 847 bp / 100% | + (5 bp) | -- | Retrotrans_gag-CCHC-RVP_2-RT-Int(GPY/F)-chromo | |  |
|  | Chr/Puccy2 | **SpoRosTy3-3** (1) | + | ND^5^ | 1003 bp^5^ / 100% | --^5^ | -- | Retrotrans_gag-CCHC-RVP_2-RT-Int(GPY/F)-chromo | |  |
|  | Chr/Tcn1 | SpoRosTy3-4 (1) | -- | ND^5^ | -- | -- | -- |  |  |  |
| Pg | Chr/MarY1 | PucGraTy3-1 (36) | -- | 6633 | 183 bp / 97.8% | -- | -- | Retrotrans_gag-CCHC | RVP_2-RT-Int(GPY/F)-chromo |  |
|  | Chr/Laccy1 | **PucGraTy3-2** (5) | + | 6124 | 265 bp / 79.8% | -- | -- | Retrotrans_gag-RVP_2-RT-Int(GPY/F)-chromo | |  |
|  | Chr/Laccy1 | **PucGraTy3-3** (2) | + | 6383 | 497-483 bp / 97% | + (5 bp) | -- | Retrotrans_gag-RVP_2-RT-Int(HHCC,GPY/F)-chromo | |  |
|  | Chr/Laccy1 | **PucGraTy3-4** (1) | + | 6288 | 475 bp / 99.4% | + (5 bp) | -- | Retrotrans_gag-RVP_2-RT-Int(GPY/F)-chromo | |  |
|  | Chr/Laccy1 | **PucGraTy3-5** (6) | + | 6209 | 140 bp / 100% | -- | -- | Retrotrans_gag-RVP_2-RT-Int(HHCC,GPY/F)-chromo | |  |
|  | Chr/Laccy1 | PucGraTy3-6 (2) | -- | 6666 | 504 bp / 99.4% | + (5 bp) | -- | Retrotrans_gag-RVP_2-RT-Int(GPY/F)-chromo | |  |
|  | Chr/Laccy1 | **PucGraTy3-7** (2) | + | 5698 | 436 bp / 99.8% | + (5 bp) | -- | Retrotrans_gag-RVP_2-RT-Int(HHCC,GPY/F)-chromo | |  |
|  | Chr/Laccy1 | **PucGraTy3-8** (4) | + | 5705 | 436 bp / 99.8% | + (5 bp) | -- | RVP_2-RT-Int(HHCC,GPY/F)-chromo | |  |
|  | Chr/Laccy1 | **PucGraTy3-9** (8) | + | 6673 | 552 bp / 100% | + (5 bp) | -- | Retrotrans_gag-RVP_2-RT-Int(HHCC,GPY/F)-chromo | |  |
|  | Chr/Laccy1 | **PucGraTy3-10** (28) | + | 6319 | 463 bp / 100% | + (5 bp) | -- | Retrotrans_gag-RVP_2-RT-Int(HHCC,GPY/F)-chromo | |  |
|  | Chr/Puccy1 | **PucGraTy3-11** (14) | + | 6119 | 351 bp / 100% | + (3bp) | -- | CCHC | RVP_2-RT-Int(GPY/F) |  |
|  | Chr/Puccy1 | **PucGraTy3-12** (4) | + | 5934 | 426 bp / 100% | + (5 bp) | -- | Retrotrans_gag-CCHC-RT-RNH-Int(GPY/F) | |  |
|  | Chr/Tcn2 | **PucGraTy3-13** (32) | + | 5724 | 357 bp / 99.7% | + (5 bp) | -- | RVP-RT-RNH-Int |  |  |
|  | Ylt1 | PucGraTy3-14 (12) | -- | 9599 | 778 bp / 99.7% | + (5 bp) | -- | CCHC-RVP_2 | RT-Int(HHCC,GPY/F) | add. antisense ORF - unknown |
|  | Ylt1 | **PucGraTy3-15** (27) | + | 10565 | 1064 bp / 100% | + (5 bp) | -- | RVP_2 | RT-Int(GPY/F) |  |
|  | Ylt1 | **PucGraTy3-16** (10) | + | 9569 | 755 bp / 98.1% | -- | -- | RVP_2-RT-Int(GPY/F) | |  |
|  | Ylt1 | PucGraTy3-17 (2) | -- | 10835 | 816 bp / 99.1% | + (4 bp) | -- | RVP | RT-Int(GPY/F) |  |

**Additional Table S1.** **(continued).** Novel Gypsy LTR retrotransposons from Fungi detected in present study, their copy number and putative structure including predicted enzymatic domains (for the intact element or retrotransposons carried long coding regions)

| Sp.^1^ | type^2^/clade | element  (copies^3^) | IE^4^ | length  (bp) | LTRs  length / homology | TSD (length) | Putative solo LTRs (number) | Gag domains^6^ | Pol domains^6^ | Additional ORFs (if present) |
| --- | --- | --- | --- | --- | --- | --- | --- | --- | --- | --- |
| Pg | Ylt1 | **PucGraTy3-18** (11) | + | 9369 | 748-749 bp / 98.3% | -- | -- | Neisseria_TspB[pfam05616] ^7^-RVP_2 | RT-Int(GPY/F) | add. antisense ORF - unknown |
|  | Ylt1 | **PucGraTy3-19** (4) | +? | 13535 | 1436 bp / 95.6% | + (5 bp) | -- | RVP | RT-Int(HHCC,GPY/F) |  |
|  | Ylt1 | **PucGraTy3-20** (3) | + | 12345 | 1131 bp / 99.6% | + (4 bp) | -- | RVP | RT-Int(HHCC,GPY/F) |  |
|  | Ylt1 | PucGraTy3-21 (3) | +? | ND^5^ | 349^5^ bp / 98.3% | --^5^ | -- | RVP | RT-Int(GPY/F) |  |
|  | Ylt1 | **PucGraTy3-22** (7) | + | 9844 | 777-764 bp / 97.8% | -- | -- |  | RT-Int(HHCC) |  |
|  | Ylt1 | **PucGraTy3-23** (16) | + | 11787 | 1990 bp / 99.9% | + (4 bp) | -- | RVP_2-RT-Int(HHCC,GPY/F) | |  |
|  | Ylt1 | **PucGraTy3-24** (12) | + | 11050 | 1109 bp / 99.8% | + (4 bp) | -- | RVP-RT-Int(GPY/F) | |  |
|  | Ylt1 | PucGraTy3-25 (2) | -- | 10759 | 764-785 bp / 97.3% | -- | -- | CCHC-RVP-RT-Int(GPY/F) -Restriction_endonuclease_like[cd01038] ^7^ | |  |
|  | Ylt1 | **PucGraTy3-26** (13) | + | 11694 | 908-910 bp / 96.1% | + (5 bp) | -- | CCHC-RVP_2 | RT-Int(GPY/F)-Restriction_endonuclease  _like[cd01038] ^7^ |  |
|  | Ylt1 | **PucGraTy3-27** (23) | + | 12077 | 970-974 bp / 98.8% | + (4 bp) | -- | CCHC-RVP_2 | RT-Int(GPY/F) |  |
|  | Chr/Puccy2 | **PucGraTy3-28** (29) | + | 9766 | 1419 bp / 99.9% | + (7 bp) | -- | CCHC-RT-Int(GPY/F)-chromo | |  |
|  | Chr/Puccy2 | **PucGraTy3-29** (27) | + | 9841 | 1458 bp /100% | + (5 bp) | -- | CCHC-RT-RNH-Int(GPY/F)-chromo | |  |
|  | Chr/Puccy2 | **PucGraTy3-30** (23) | + | 9648 | 977-976 bp / 97.4% | -- | -- | CCHC-RVP-RT-Int(GPY/F)-chromo | |  |
|  | Chr/Puccy2 | **PucGraTy3-31** (2) | + | 9435 | 1114-1088 bp / 97.3% | + (5 bp) | -- | CCHC-RT-Int(GPY/F)-chromo | |  |
|  | Chr/Puccy2 | **PucGraTy3-32** (5) | + | 9922 | 1516-1517 bp / 99.9% | + (5 bp) | -- | CCHC-RT-Int(GPY/F)-chromo | |  |
|  | Chr/Puccy2 | **PucGraTy3-33** (27) | + | 9323 | 773-791 bp / 97.3% | -- | -- | CCHC-RVP-RT-Int(GPY/F)-chromo | |  |
|  | Chr/Puccy2 | **PucGraTy3-34** (4) | + | 7745 | 419 bp / 100% | + (4 bp) | -- | CCHC-RT-Int(GPY/F)-chromo | |  |
|  | Chr/Puccy2 | **PucGraTy3-35** (70) | + | 9942 | 1087 bp / 99.6% | + (5 bp) | -- | CCHC-RVP-RT-Int(GPY/F) | |  |
|  | Chr/Puccy2 | PucGraTy3-36 (10) | -- | 10463 | 1323 bp / 99.6% | -- | -- | CCHC-RT-Int(GPY/F)-chromo | |  |
|  | Chr/Puccy2 | PucGraTy3-37 (3) | -- | 8974 | 790-777 bp / 86.2% | -- | -- | CCHC-RT-Int(GPY/F)-chromo | |  |
| Bden | Chr/Tcn1 | **BatDenTy3-1** (1) | + | 5440 | 265-314 bp / 81.5% | -- | -- | Retrotrans_gag-CCHC | RVP_2-RT-Int(GPY/F)-chromo |  |

LTR retrotransposons represented by at least one putatively intact copy are highlighted by bold. ND – not determined.

# ^1^Species names: Cg – *Chaetomium globosum* CBS 148.51; Fo – *Fusarium oxysporum* 4286 FGSC 4286; Fv – *Fusarium verticillioides* 7600; Nh – *Nectria haematococca* MPVI; Pa – *Podospora anserina* S mat+; Tr – *Trichoderma reesei* QM6a; Tv – *Trichoderma virens* Gv29-8; Ac – *Aspergillus clavatus* NRRL1; Ani – *Aspergillus niger* ATCC1015; At – *Aspergillus terreus* NIH2624; Ci – *Coccidioides immitis* RS; Hc – *Histoplasma capsulatum* NAm1; Ur – *Uncinocarpus reesii* 1704; Ss – *Sclerotinia sclerotiorum* 1980; Bc – *Botrytis cinerea* B05.10; Abr – *Alternaria brassicicola* ATCC 96866; Ptr – *Pyrenophora tritici-repentis*; Sn – *Stagonospora nodorum* SN15; Ab – *Amanita bisporigera*; Cc – *Coprinus cinereus* Okayama7#130; Lb – *Laccaria bicolor* S238N; Pp – *Postia placenta* MAD-698; Sr - *Sporobolomyces roseus*; Pg - *Puccinia graminis f. sp. tritici*.

^2^Three types of elements were found: Chr – chromodomain-containing LTR retrotransposons, Ylt1-like retrotransposons and new group which was named SN1006.

^3^Number of copies per haploid genome is given according HMMER search results for fungi, according to the BLAST (blastp or blastx) for plants and *Postia placenta* MAD-698 [1] – corresponding RT region was used as query.

^4^IE – intact full-length element if detected.

^5^Copies have 5’ or/and 3’ truncations and/or long indels and/or contain polyN stretches.

# ^6^The best-scoring hits in CDD database [2] available at NCBI [3]: Retrotrans_gag – pfam03732; CCHC – pfam00098; RVP – pfam00077; RVP_2 – pfam08284; RT – cd01647; RNH – pfam00075; Int – pfam00665; HHCC – pfam09337; chromo – cd00024.

^7^Additional domains not characteristic for LTR retrotransposons in order of appearance:

**Herpes_UL51.** Herpesvirus UL51 protein. The best-scoring hit on this query sequence is by member pfam04540:

**CD Length:** 239  **Bit Score:** 31.60  **E-value:** 1.8

10 20 30 40 50 60

....*....|....*....|....*....|....*....|....*....|....*....|..

ChaGloTy3-5a [gag-pol] 901 AVAEHDLGLRAPDAPMvvnynGTSSTPVSDYVYQLTQ-GATVDPPAGDQAPEEPDLPVVPEK 961

pfam04540 150 AVLERALGLDAQPQDA-----STRALAAGTGVLNLLRmGLVPPPDLKDPPALVEVIDVLPEK 206

**RT_like**: Reverse transcriptase (RT, RNA-dependent DNA polymerase)_like family. An RT gene is usually indicative of a mobile element such as a retrotransposon or retrovirus. RTs occur in a variety of mobile elements, including retrotransposons, retroviruses, group II introns, bacterial msDNAs, hepadnaviruses, and caulimoviruses. These elements can be divided into two major groups. One group contains retroviruses and DNA viruses whose propagation involves an RNA intermediate. They are grouped together with transposable elements containing long terminal repeats (LTRs). The other group, also called poly(A)-type retrotransposons, contain fungal mitochondrial introns and transposable elements that lack LTRs. The best-scoring hit on this query sequence is by member cd01644:

**CD Length:** 213  **Bit Score:** 28.33  **E-value:** 7.0

10 20 30 40 50 60

....*....|....*....|....*....|....*....|....*....|....*....|

BotCinTy3-4 [add. ORF] 540 LWREVAKQRMDQEYRLVTVPF-----PALIIQAEEDFDRTFVHGNA-----RDLYtVSDY 589

cd01644 86 LWRKDGDEPKPIEYRMTVVPFgaasaPFLANRALKQHAEDHPHEAAakiikRNFY-VDDI 144

**Birna_VP4.** Birnavirus VP4 protein. The best-scoring hit on this query sequence is by member pfam01768:

**CD Length:** 259  **Bit Score:** 31.38  **E-value:** 2.7

10 20 30 40 50 60

....*....|....*....|....*....|....*....|....*....|....*....|..

**LacBicTy3-9 [gag-pol]** 1116 RDTAPPARKPTHQVSIEEMEDEdvvaarIKPTSPRHllIPMDGIGDPEENYEPNRSEDIKTA 1177

pfam01768 176 KDFAGPIMGPSGQLAISLLDND------IRPGVPRM--VFTGEIANDESTIEPVCGVDIKLI 229

**dUTPase.** dUTPase The best-scoring hit on this query sequence is by member TIGR00576:

**CD Length:** 141  **Bit Score:** 78.80  **E-value:** 2e-16

10 20 30 40 50 60 70 80

....*....|....*....|....*....|....*....|....*....|....*....|....*....|....*....|

PosPlaTy3-4 [pol] 1247 PKRKTDTAAGFELYASQNQEVASNATLQIRTGIRIQLPVGTYGRITP-VGNPLAKGIQAIE--GIIEWGDPAEVKVVVTN 1323

TIGR00576 13 PTYATEGAAGYDLYAAEDVTIPPGERALVPTGIAIELPDGYYGRVAPrSGLALKHGVTIDNspGVIDADYRGEIKVILIN 92

90 100

....*....|....*....|

PosPlaTy3-4 [pol] 1324 TGPSELKIAKGDPIAQLIIE 1345

TIGR00576 93 LGKEDFTVKKGDRIAQLVVE 112

**Neisseria_TspB.** Neisseria meningitidis TspB protein The best-scoring hit on this query sequence is by member pfam05616:

**CD Length:** 508  **Bit Score:** 34.28  **E-value:** 0.12

10 20 30 40 50 60 70 80

....*....|....*....|....*....|....*....|....*....|....*....|....*....|....*....|

PucGraTy3-18 [gag] 30 QILP-PQLPPHQYYNPGTtfyQP-----PAHNPAFNPGPMRNP----QPSESDDLNpleiqeplyPQGTPSQQTGPappv 99

pfam05616 314 QVIPrPDLTPGSAEAPNA---QPlpevsPAENPANNPNPNENPgtspNPEPDPDLN---------PDANPDTDGQP---- 377

90 100

....*....|....*....|....*

PucGraTy3-18 [gag] 100 rntATQPTHPLEPKKPTPIHFLERE 124

pfam05616 378 ---GTRPDSPAVPDRPNGRDGKDGK 399

**Restriction_endonuclease_like.** Superfamily of nucleases including Short Patch Repair (Vsr) Endonucleases, archaeal Holliday junction resolvases, MutH methy-directed DNA mismatch-repair endonucleases, and catalytic domains of many restriction endonucleases, such as EcoRI, BamHI, and FokI The best-scoring hit on this query sequence is by member cd01038:

**CD Length:** 108  **Bit Score:** 33.67  **E-value:** 0.39

10 20 30 40 50 60

....*....|....*....|....*....|....*....|....*....|....*....|....*...

PucGraTy3-25 [gag-pol] 1901 RRMAARLRQPLDPGELVLvynksledqWGKLFSHRWNGpFKIKKQLPKGSYILE----------ELDG 1958

cd01038 3 RDRARELRRNQTDAERLL---------WQELRRRRLNG-FKFRRQAPIGRYIVDfacpeaklvvELDG 60

# References

1. **The DOE Joint Genome Institute** [http://genome.jgi-psf.org/Pospl1/Pospl1.home.html]

2. Marchler-Bauer A, Anderson JB, Chitsaz F, et al. (25 co-authors): **CDD: specific functional annotation with the Conserved Domain Database.** *Nucleic Acids Res* 2009, **37**:D205-210.

3. **National Center for Biotechnology Information** [http://www.ncbi.nlm.nih.gov]
