# Supplementary material for: Evolutionary genomics revealed interkingdom distribution of Tcn1-like chromodomain-containing Gypsy LTR retrotransposons among fungi and plants
Source: BMC Genomics. 2010 Apr 8;11:231. doi: 10.1186/1471-2164-11-231 (PMC2864245; doi:10.1186/1471-2164-11-231)
Supplement: Additional file 2 — Phylogenetic analysis of dUTPase. Neighbor-Joining phylogenetic tree reconstructed based on dUTPase amino acid sequences from eukaryotes, viruses, and dUTPase domains from CHD-containing Gypsy LTR retrotransposons. [file 1471-2164-11-231-S2.DOC]

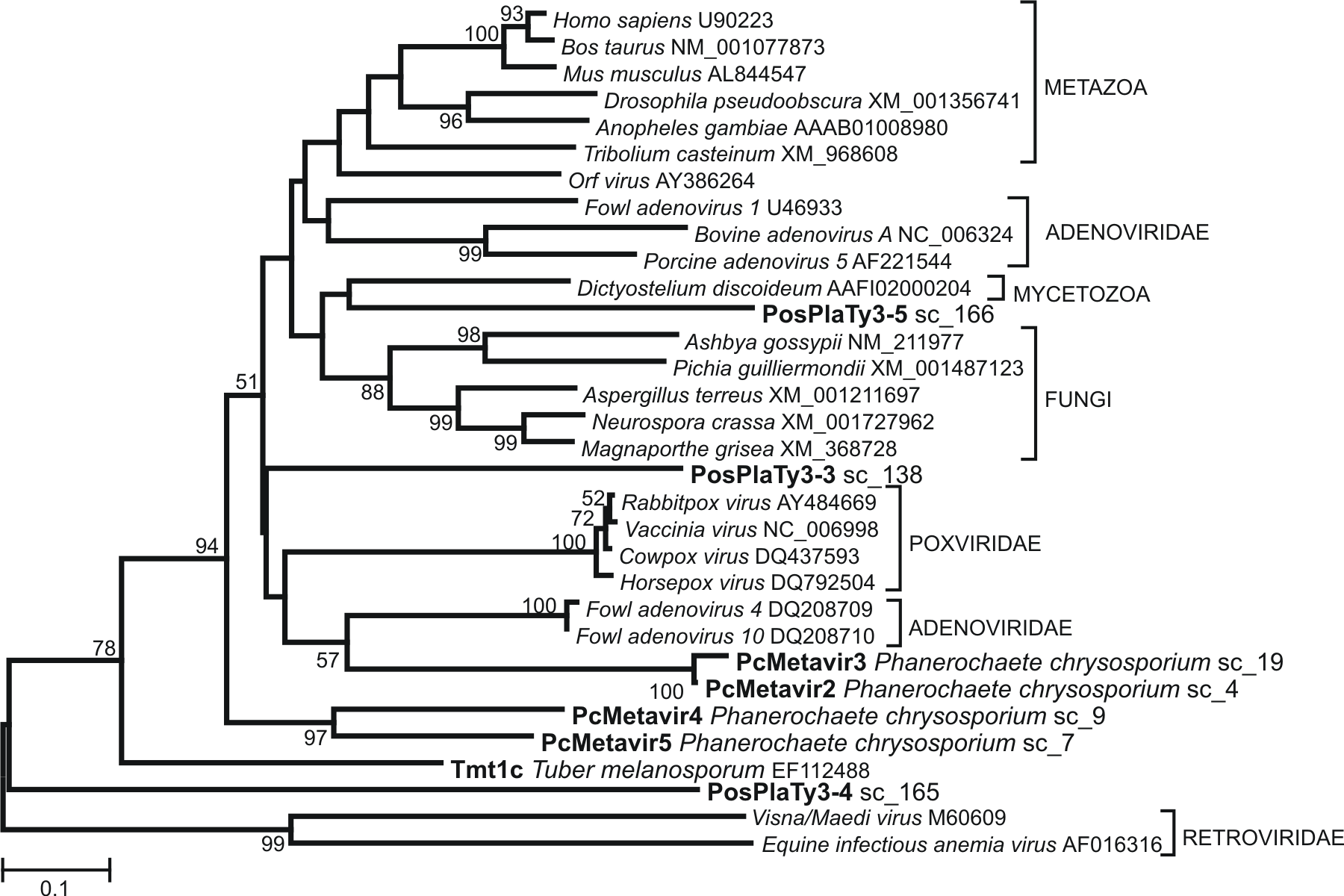
**Additional file 2.** Phylogenetic analysis (NJ) of amino acid sequences of dUTPase of eukaryotes, viruses, and dUTPase domains from CHD-containing Gypsy LTR retrotransposons (highlighted by bold) from *Phanerochaete chrysosporium* RP-70 (PcMetavir2-5 [1]), *Tuber melanosporum* (Tmt1c [2]), and newly identified PosPlaTy3-3, 4, 5 from *Postia placenta* MAD-698*.* Bootstraps less than 50% are not shown. Accession numbers in GenBank are given. Genomic sequences of *Phanerochaete chrysosporium* RP-70 and *Postia placenta* MAD-698 are available at DOE Joint Genome Institute [3].

**References**

1. Novikova OS, Blinov AG: **dUTPase-containing Metaviridae LTR retrotransposons from the genome of Phanerochaete chrysosporium (Fungi: Basidiomycota).** *Dokl Bioch Bioph* 2008, **420**:146-149.

2. Riccioni C, Rubini A, Belfiori B, Passeri V, Paolocci F, Arcioni S: **Tmt1: the first LTR-retrotransposon from a Tuber spp.** *Curr Genet* 2008, **53**:23-34.

3. **The DOE Joint Genome Institute** [http://www.jgi.doe.gov]
