# Supplementary material for: Evolutionary genomics revealed interkingdom distribution of Tcn1-like chromodomain-containing Gypsy LTR retrotransposons among fungi and plants
Source: BMC Genomics. 2010 Apr 8;11:231. doi: 10.1186/1471-2164-11-231 (PMC2864245; doi:10.1186/1471-2164-11-231)

**Additional file 4.** Neighbor-joining (NJ) phylogenetic tree based on RT nucleotide sequences of Athila-like LTR retrotransposons including newly described elements (highlighted by bold) from monilophytes and lycophyte plants: *Matteuccia struthiopteris* (L.) Tod*.* (MattStrutAthila-1), *Athyrium distentifolium* Tausch ex Opiz (AthyDistAthila-1), *Athyrium sinense* Rupr. (AthySin Athila-1,2, and 3), *Athyrium monomachii* (Kom.) Kom. (AthyMonAthila-1), *Asplenium viride* Huds. (AspVirAthila-1), *Pteridium aquilinum* (L.) Kuhn (PteAquAthila-1), *Pyrrosia lingua* (Thunb.) Farw. (PyrLingAthila-1), and *Lycopodium annotinum* L. (LycAnnAthila-1) as well as new LTR retrotransposons from *Ginkgo biloba* L. (GinBilAthila-1,2, and 3), *Pinus radiata* D. Don (PinRadAthila-1 and PinRadAthila-2), *Ephedra distachya* L. (EphDistAthila-1), *Peperomia caperata* Yunck. (PepCapAthila-1 and PepCapAthila-2), and *Pelargonium zonale* (L.) L'Hér*.* ex Ait. (PelZonAthila-1). Statistical support was evaluated by bootstrapping (1000 replications); nodes with bootstrap values over 50% are shown. The name of the host species and accession number are indicated for LTR retrotransposons taken from GenBank. The newly identified LTR retrotransposons are available in GenBank under accession numbers GQ443436- GQ443445 and AY959294- AY959313.

Total DNA of stiff clubmoss *Lycopodium annotinum* and pine *Pinus radiata* was provided by Royal Botanic Gardens, Kew, England. Novel Athila-like LTR retrotransposons sequences from monilophytes and lycophyte were obtained additionally to the targeted CHD-containing LTR retrotransposons by PCR amplification using primers GyRT1 = 5'-MRNATGTGYGTNGAYTAYMG-3' and ty3-A = 5'-AATTCGCTGCCGCTAAGATNARNADRTCRTC-3', where M = A + C, Y = C + T, R = A + G, D = A + G + T and N = A + G + C + T. Novel Athila-like LTR retrotransposons from seed plants listed above were obtained in our previous survey by PCR amplification with following primers: ty3-S = 5’- AATTCTGGCACTTTTCGACTNTGYRTNGAYTA -3’ and ty3-A.


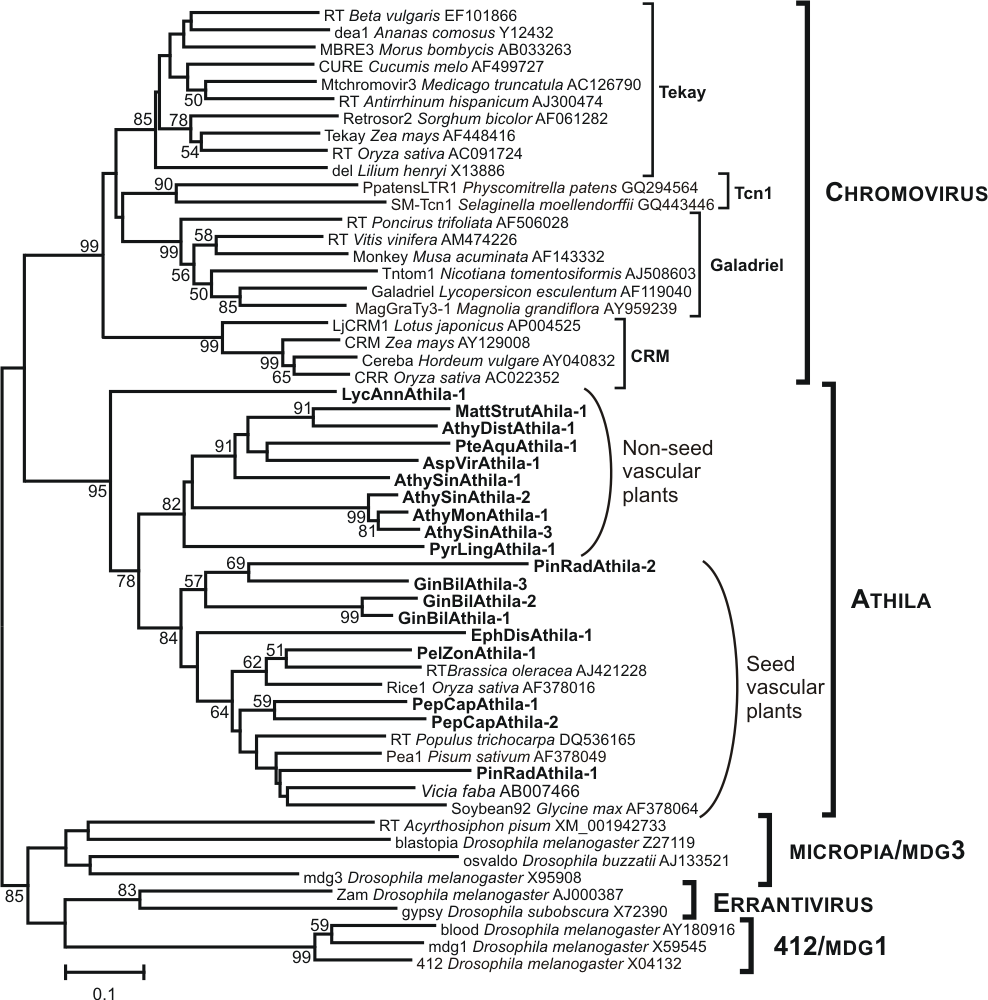

Supplement: Additional file 4 — Phylogenetic analysis of Athila-like LTR retrotransposons. Neighbor-joining (NJ) phylogenetic tree based on RT nucleotide sequences of Athila-like LTR retrotransposons including newly described elements. [file 1471-2164-11-231-S4.DOC]
