# Supplementary material for: Evolutionary genomics revealed interkingdom distribution of Tcn1-like chromodomain-containing Gypsy LTR retrotransposons among fungi and plants
Source: BMC Genomics. 2010 Apr 8;11:231. doi: 10.1186/1471-2164-11-231 (PMC2864245; doi:10.1186/1471-2164-11-231)
Supplement: Additional file 5 — List of fungal species, genomes of which were analyzed. Table contained the list of fungal species, genomes of which were analyzed in silico in the present study and the sources of genomic sequences. [file 1471-2164-11-231-S5.DOC]

| Species | Organization: Web link |
| --- | --- |
| *Chaetomium globosum* CBS 148.51 | Broad Institute: http://www.broad.mit.edu/annotation/genome/chaetomium_globosum/Home.html |
| *Fusarium oxysporum* 4286 FGSC | Broad Institute: http://www.broad.mit.edu/annotation/genome/fusarium_group/MultiHome.html |
| *Fusarium verticillioides* 7600 | Broad Institute: http://www.broad.mit.edu/annotation/genome/fusarium_group/MultiHome.html |
| **Nectria haematococca** MPVI | http://genome.jgi-psf.org/Necha2/Necha2.home.html |
| *Trichoderma reesei* QM6a | JGI: http://genome.jgi-psf.org/Trire2/Trire2.home.html |
| *Trichoderma virens* Gv29-8 | JGI: http://genome.jgi-psf.org/Trive1/Trive1.home.html |
| *Podospora anserina* S mat+ | CNRS: http://podospora.igmors.u-psud.fr/ |
| *Aspergillus clavatus* NRRL 1 | Broad Institute: http://www.broad.mit.edu/annotation/genome/aspergillus_terreus/MultiHome.html |
| *Aspergillus niger* ATCC1015 | JGI: http://genome.jgi-psf.org/Aspni1/Aspni1.home.html |
| *Aspergillus terreus* NIH2624 | Broad Institute: http://www.broad.mit.edu/annotation/genome/aspergillus_group/GenomesIndex.html |
| *Coccidioides immitis* RS | Broad Institute: http://www.broad.mit.edu/annotation/genome/coccidioides_group/MultiHome.html |
| *Histoplasma capsulatum* NAm1 | Broad Institute: http://www.broad.mit.edu/annotation/genome/histoplasma_capsulatum/Home.html |
| *Uncinocarpus reesii* 1704 | Broad Institute: http://www.broad.mit.edu/annotation/genome/uncinocarpus_reesii/Home.html |
| *Sclerotinia sclerotiorum* 1980 | Broad Institute: http://www.broad.mit.edu/annotation/genome/sclerotinia_sclerotiorum/Home.html |
| *Botrytis cinerea* B05.10 | Broad Institute: http://www.broad.mit.edu/annotation/genome/botrytis_cinerea/Home.html |
| *Alternaria brassicicola* ATCC 96866 | JGI: http://genomeportal.jgi-psf.org/Altbr1/Altbr1.home.html |
| *Pyrenophora tritici-repentis* | Broad Institute: http://www.broad.mit.edu/annotation/genome/pyrenophora_tritici_repentis/Home.html |
| *Stagonospora nodorum* SN15 | JGI: http://genomeportal.jgi-psf.org/Stano1/Stano1.home.html |
| *Amanita bisporigera* | https://www.msu.edu/user/hallenhe/amanita.htm |
| *Coprinus cinereus* okayama7#130 | Broad Institute: http://www.broad.mit.edu/annotation/genome/coprinus_cinereus/Home.html |
| *Laccaria bicolor* S238N | JGI: http://genome.jgi-psf.org/laccaria/ |
| *Postia placenta* MAD-698 | JGI: http://genome.jgi-psf.org/Pospl1/Pospl1.home.html |
| *Sporobolomyces roseus* | JGI: http://genome.jgi-psf.org/Sporo1/Sporo1.home.html |
| *Puccinia graminis f. sp. tritici* | Broad Institute: http://www.broad.mit.edu/annotation/genome/puccinia_graminis |
| *Batrachochytrium dendrobatidis* JEL423 | Broad Institute: http://www.broad.mit.edu/annotation/genome/batrachochytrium_dendrobatidis |

**Additional Table S2.** List of species genomes of which were analyzed *in silico* in present study and the sources of genomic sequences
