# Supplementary material for: Evolutionary genomics revealed interkingdom distribution of Tcn1-like chromodomain-containing Gypsy LTR retrotransposons among fungi and plants
Source: BMC Genomics. 2010 Apr 8;11:231. doi: 10.1186/1471-2164-11-231 (PMC2864245; doi:10.1186/1471-2164-11-231)
Supplement: Additional file 6 — Novel Gypsy LTR retrotransposons from Fungi. Table contained the list of novel Gypsy LTR retrotransposons from Fungi detected in present study and their accession numbers. [file 1471-2164-11-231-S6.DOC]

**Additional Table S3.** Novel non-LTR retrotransposons from Fungi detected in this study and their accession numbers in databases

| Species | element | database: genomic scaffold/protein  and/or database: contig [location of HMM signal] |
| --- | --- | --- |
| *Chaetomium globosum* | ChaGloTy3-1 | GenBank: XM_001229222/XP_001229223 |
|  | ChaGloTy3-2 | GenBank: XM_001222219/XP_001222220 |
|  | ChaGloTy3-3 | GenBank: XM_001229594/XP_001229595. |
|  | ChaGloTy3-4 | GenBank: XM_001222906/XP_001222907 |
|  | ChaGloTy3-5a  ChaGloTy3-5b | GenBank: XM_001228540/XP_001228541  GenBank: XM_001226319/XP_001226320 |
|  | ChaGloTy3-6 | GenBank: XM_001230211/XP_001230212 |
|  | ChaGloTy3-7 | GenBank: XM_001223152/XP_001223153 |
|  | ChaGloTy3-8 | GenBank: XM_001224270/XP_001224271 |
| *Fusarium oxysporum* | FusOxyTy3-1 | Broad Institute: Supercontig 31 [265284..267395] |
|  | FusOxyTy3-2 | Broad Institute: Supercontig 17 [1025765..1027876] |
|  | FusOxyTy3-3 | Broad Institute: Supercontig 14 [159070..160920] |
| *Fusarium verticillioides* | FusVerTy3-1 | Broad Institute: Supercontig 16 [10078..12339] |
|  | FusVerTy3-2 | Broad Institute: Supercontig 1 [654494..656272] |
| *Nectria haematococca* | NecHaemTy3-1 | JGI: scaffold_19 [647885..649822] |
|  | NecHaemTy3-2 | JGI: scaffold_59 [37267..39510] |
|  | NecHaemTy3-3 | JGI: scaffold_9 [606565..608946] |
|  | NecHaemTy3-4 | JGI: scaffold_24 [8256..10382] |
|  | NecHaemTy3-5 | JGI: scaffold_20 [1931664..1933754] |
|  | NecHaemTy3-6 | JGI: scaffold_107 [30722..32815] |
| *Podospora anserina* | PodAnsTy3-1 | GenBank: CU633867 |
|  | PodAnsTy3-2 | GenBank: EU697464 |
|  | PodAnsTy3-3 | GenBank: EU697468 |
| *Trichoderma reesei* | TrichReeTy3-1 | JGI: scaffold_1 [3174430..3176460] |
|  | TrichReeTy3-2 | JGI: scaffold_27 [336334..338655] |
|  | TrichReeTy3-3 | JGI: scaffold_10 [129765..132233] |
|  | TrichReeTy3-4 | JGI: scaffold_38 [5755..8010] |
| *Trichoderma virens* | TrichVirTy3-1 | JGI: scaffold_16 [13069..15012] |
| *Aspergillus clavatus* | AspClaTy3-1 | Broad Institute: Supercontig 77 [961166..963391] |
|  | AspClaTy3-2 | Broad Institute: Supercontig 17 [658853..661039] |
|  | AspClaTy3-3 | Broad Institute: Supercontig 84 [266552..268471] |
|  | AspClaTy3-4 | Broad Institute: Supercontig 86 [2603706..2605886] |
|  | AspClaTy3-5 | Broad Institute: Supercontig 79 [1111197..1113380] |
| *Aspergillus niger* | AspNigTy3-1 | GenBank: AM270026  Broad Institute: Supercontig 2 [2842095..2843267] |
| *Aspergillus terreus* | AspTerTy3-1 | GenBank: NT_165938/XP_001218108  Broad Institute: Supercontig 13 [243312..245291] |
| *Coccidioides immitis* | CocImmTy3-1 | Broad Institute: Supercontig 1 [3222089..3224380] |
|  | CocImmTy3-2 | Broad Institute: Supercontig 4 [3002149..3004356] |
|  | CocImmTy3-3 | Broad Institute: Supercontig 4 [2605251..2607284] |
|  | CocImmTy3-4 | Broad Institute: Supercontig 6 [3413419..3415470] |
|  | CocImmTy3-5 | Broad Institute: Supercontig 6 [3369225..3371219] |
|  | CocImmTy3-6 | Broad Institute: Supercontig 5 [3447778..3449865] |
|  | CocImmTy3-7a  CocImmTy3-7b | Broad Institute: Supercontig 1 [637333..639168]  Broad Institute: Supercontig 1 [6159448..6161538] |
|  | CocImmTy3-8 | Broad Institute: Supercontig 2 [245085..247184] |

**Additional Table S3 (continued).** Novel non-LTR retrotransposons from Fungi detected in this study and their accession numbers

| Species | element | database: genomic scaffold/protein  and/or database: contig [location of HMM signal] |
| --- | --- | --- |
| *Histoplasma capsulatum* | HisCapTy3-1 | GenBank: XM_001540215/XP_001540265  Broad Institute: Supercontig 1 [1638925..1641279] |
|  | HisCapTy3-2 | Broad Institute: Supercontig 3 [625391..627733] |
|  | HisCapTy3-3 | Broad Institute: Supercontig 5 [1285335..1287434] |
|  | HisCapTy3-4 | GenBank: XM_001538690/XP_001538740  Broad Institute: Supercontig 9 [25345..27612] |
|  | HisCapTy3-5 | Broad Institute: Supercontig 9 [983375..984547] |
|  | HisCapTy3-6 | GenBank: XM_001541811/XP_001541861  Broad Institute: Supercontig 7 [1431933..1434233] |
|  | HisCapTy3-7 | Broad Institute: Supercontig 4 [573469..575415] |
|  | HisCapTy3-8 | Broad Institute: Supercontig 1 [1129834..1132020] |
|  | HisCapTy3-9 | GenBank (partial): XM_001537035/XP_001537085  Broad Institute: Supercontig 10 [1009863..1011761] |
|  | HisCapTy3-10 | GenBank: XM_001539008/ XP_001539058  Broad Institute: Supercontig 6 [1100209..1102356] |
|  | HisCapTy3-11 | Broad Institute: Supercontig 2 [37337..39439] |
| *Uncinocarpus reesii* | UncReeTy3-1 | Broad Institute: Supercontig 3 [3810230..3812323] |
|  | UncReeTy3-2 | Broad Institute: Supercontig 4 [2557425..2559542] |
|  | UncReeTy3-3 | Broad Institute: Supercontig 3 [3782154..3784202] |
|  | UncReeTy3-4 | Broad Institute: Supercontig 2 [2070404..2072437] |
|  | UncReeTy3-5 | Broad Institute: Supercontig 1 [35459..37741] |
|  | UncReeTy3-6 | Broad Institute: Supercontig 1 [43119..45296] |
| *Sclerotinia sclerotiorum* | ScleSclerTy3-1 | Broad Institute: Supercontig 6 [1674668..1676707] |
|  | ScleSclerTy3-2 | Broad Institute: Supercontig 1 [2401389..2403407] |
|  | ScleSclerTy3-3 | Broad Institute: Supercontig 6 [169905..171968] |
|  | ScleSclerTy3-4 | Broad Institute: Supercontig 4 [1692626..1694698] |
| *Botrytis cinerea* | BotCinTy3-1 | GenBank: XM_001545906/XP_001545956  Broad Institute: Supercontig 183 [39325..41355] |
|  | BotCinTy3-2 | GenBank: XM_001552430/XP_001552480  Broad Institute: Supercontig 56 [216575..218830] |
|  | BotCinTy3-3 | GenBank: XM_001548791/XP_001548841  Broad Institute: Supercontig 76 [149262..151409] |
|  | BotCinTy3-4 | GenBank: XM_001559456/XP_001559506  Broad Institute: Supercontig 98 [77242..79362] |
| *Alternaria brassicicola* | AltBraTy3-1 | WU GSC: Contig1.100 [103..2361] |
|  | AltBraTy3-2 | WU GSC: Contig5.76 [21527..23647] |
|  | AltBraTy3-3 | WU GSC: Contig9.4 [19243..20529] |
|  | AltBraTy3-4 | WU GSC: Contig2.232 [29746..31787] |
|  | AltBraTy3-5 | WU GSC: Contig0.191 [63097..64678] |
|  | AltBraTy3-6 | WU GSC: Contig9.4 [25429..27579] |
| *Pyrenophora tritici-repentis* | PyrTriTy3-1 | Broad Institute: Supercontig 4 [854996..856894] |
|  | PyrTriTy3-2 | Broad Institute: Supercontig 12 [24175..26292] |
|  | PyrTriTy3-3 | Broad Institute: Supercontig 2 [3277605..3279860] |
|  | PyrTriTy3-4 | Broad Institute: Supercontig 9 [590204..592459] |
|  | PyrTriTy3-5 | Broad Institute: Supercontig 1 [4893399..4895747] |
|  | PyrTriTy3-6 | Broad Institute: Supercontig 26 [96995..99460] |
|  | PyrTriTy3-7 | Broad Institute: Supercontig 19 [346025..348334] |
|  | PyrTriTy3-8 | Broad Institute: Supercontig 4 [1290478..1292601] |

**Additional Table S3 (continued).** Novel non-LTR retrotransposons from Fungi detected in this study and their accession numbers

| Species | element | database: genomic scaffold/protein  and/or database: contig [location of HMM signal] |
| --- | --- | --- |
| *Stagonospora nodorum* | StaNodTy3-1 | JGI: scaffold_4 [5548..7533] |
|  | StaNodTy3-2 | JGI: scaffold_49 [94816..97101] |
|  | StaNodTy3-3 | JGI: scaffold_55 [26336..28393] |
|  | StaNodTy3-4 | JGI: scaffold_67 [6387..8267] |
|  | StaNodTy3-5 | JGI: scaffold_19 [808455..810926] |
| *Amanita bisporigera* | AmaBisTy3-1 | GenBank: GQ294561 |
| *Coprinus cinereus* | CopCinTy3-1 | GenBank: XM_001834840/XP_001834892  Broad Institute: supercontig 12 [852062..854125] |
|  | CopCinTy3-2 | GenBank: XM_001834926/XP_001834978  Broad Institute: supercontig 1 [4134838..4136739] |
|  | CopCinTy3-3 | GenBank: XM_001838010/XP_001838062  Broad Institute: supercontig 6 [1527266..1529329] |
|  | CopCinTy3-4 | GenBank: XM_001839944/XP_001839996  Broad Institute: supercontig 2 [3223361..3225421] |
|  | CopCinTy3-5 | GenBank: XM_001834739/XP_001834791  Broad Institute: supercontig 2 [632475..634481] |
|  | CopCinTy3-6 | GenBank: XM_001834772/XP_001834824  Broad Institute: supercontig 12 [416074..418107] |
|  | CopCinTy3-7 | GenBank: XM_001829959/XP_001830011  Broad Institute: supercontig 3 [841518..843569] |
|  | CopCinTy3-8 | GenBank: XM_001828110/XP_001828162  Broad Institute: supercontig 1 [97173..99305] |
|  | CopCinTy3-9 | GenBank: XM_001840214/XP_001840266  Broad Institute: supercontig 10 [752042..754324] |
|  | CopCinTy3-10 | GenBank: XM_001835230/XP_001835282  Broad Institute: supercontig 11 [170267..172318] |
|  | CopCinTy3-11 | GenBank: XM_001841125/XP_001841177  Broad Institute: supercontig 2 [188327..190810] |
|  | CopCinTy3-12 | GenBank: XM_001835305/XP_001835357  Broad Institute: supercontig 12 [2094327..2096852] |
|  | CopCinTy3-13 | GenBank: XM_001839531/XP_001839583  Broad Institute: supercontig 11 [52160..54538] |
|  | CopCinTy3-14 | GenBank: XM_001838426/XP_001838478  Broad Institute: supercontig 4 [762226..764379] |
|  | CopCinTy3-15 | GenBank: XM_001841487/XP_001841539  Broad Institute: supercontig 32 [3726..5951] |
|  | CopCinTy3-16 | GenBank: XM_001840638/XP_001840690  Broad Institute: supercontig 3 [3367519..3369870] |
| *Postia placenta* | PosPlaTy3-1 | JGI: scaffold_192 [66545..68593] |
|  | PosPlaTy3-2 | JGI: scaffold_46 [117787.. 119055] |
|  | PosPlaTy3-3 | JGI: scaffold_138 [209647..211398] |
|  | PosPlaTy3-4 | JGI: scaffold_165 [192415..194451] |
|  | PosPlaTy3-5 | JGI: scaffold_166 [116365..117894] |
|  | PosPlaTy3-6 | JGI: scaffold_1655 [3856..5637] |
|  | PosPlaTy3-7 | JGI: scaffold_89 [286932.. 288047] |
|  | PosPlaTy3-8 | JGI: scaffold_112 [129052.. 130944] |
|  | PosPlaTy3-9 | JGI: scaffold_23 [304083.. 306128] |
| *Sporobolomyces roseus* | SpoRosTy3-1 | JGI: scaffold_9 [8398..6683] |
|  | SpoRosTy3-2 | JGI: scaffold_16 [4617..6707] |
|  | SpoRosTy3-3 | JGI: scaffold_1 [4037819..4039906] |
|  | SpoRosTy3-4 | JGI: scaffold_1 [77903.. 79402] |

**Additional Table S3 (continued).** Novel non-LTR retrotransposons from Fungi detected in this study and their accession numbers

| Species | element | database: genomic scaffold/protein  and/or database: contig [location of HMM signal] |
| --- | --- | --- |
| *Laccaria bicolor* | LacBicTy3-1 | JGI: scaffold_24 [453115.. 455160] |
|  | LacBicTy3-2 | JGI: scaffold_21 [575423..577123] |
|  | LacBicTy3-3 | JGI: scaffold_174 [36462..38906] |
|  | LacBicTy3-4 | JGI: scaffold_56 [218006.. 220414] |
|  | LacBicTy3-5 | JGI: scaffold_91 [33665..35776] |
|  | LacBicTy3-6 | JGI: scaffold_2 [1588979..1591105] |
|  | LacBicTy3-7 | JGI: scaffold_23 [567756..570131] |
|  | LacBicTy3-8 | JGI: scaffold_8 [406403..408835] |
|  | LacBicTy3-9 | JGI: scaffold_38 [15367.. 17679] |
|  | LacBicTy3-10 | JGI: scaffold_9 [1126007.. 1128439] |
|  | LacBicTy3-11 | JGI: scaffold_14 [706845..708653] |
|  | LacBicTy3-12 | JGI: scaffold_38 [387217..389865] |
|  | LacBicTy3-13 | JGI: scaffold_63 [197228.. 199639] |
|  | LacBicTy3-14 | JGI: scaffold_74 [95099..97801] |
|  | LacBicTy3-15 | JGI: scaffold_10 [269197:271281] |
| *Puccinia graminis f. sp. tritici* | PucGraTy3-1 | Broad Institute: Supercontig 107 [68798..70957] |
|  | PucGraTy3-2 | Broad Institute: Supercontig 3 [798427..800535] |
|  | PucGraTy3-3 | Broad Institute: Supercontig 5 [691753..693855] |
|  | PucGraTy3-4 | Broad Institute: Supercontig 41 [548160..550268] |
|  | PucGraTy3-5 | Broad Institute: Supercontig 80 [278515..280599] |
|  | PucGraTy3-6 | Broad Institute: Supercontig 12 [1433008..1435092] |
|  | PucGraTy3-7 | Broad Institute: Supercontig 134 [1596..3608] |
|  | PucGraTy3-8 | Broad Institute: Supercontig 15 [971124..973136] |
|  | PucGraTy3-9 | Broad Institute: Supercontig 1 [2068580..2070610] |
|  | PucGraTy3-10 | Broad Institute: Supercontig 5 [1205976..1207991] |
|  | PucGraTy3-11 | Broad Institute: Supercontig 37 [307776..309776] |
|  | PucGraTy3-12 | Broad Institute: Supercontig 4 [1950228..1952246] |
|  | PucGraTy3-13 | Broad Institute: Supercontig 25 [1050910..1052913] |
|  | PucGraTy3-14 | Broad Institute: Supercontig 24 [362395..364470] |
|  | PucGraTy3-15 | Broad Institute: Supercontig 18 [76106..78220] |
|  | PucGraTy3-16 | Broad Institute: Supercontig 29 [759732..761825] |
|  | PucGraTy3-17 | Broad Institute: Supercontig 4 [963851..965944] |
|  | PucGraTy3-18 | Broad Institute: Supercontig 6 [693953..696043] |
|  | PucGraTy3-19 | Broad Institute: Supercontig 52 [353236..355329] |
|  | PucGraTy3-20 | Broad Institute: Supercontig 1 [166207..168300] |
|  | PucGraTy3-21 | Broad Institute: Supercontig 46 [447219..449312] |
|  | PucGraTy3-22 | Broad Institute: Supercontig 20 [271912..273990] |
|  | PucGraTy3-23 | Broad Institute: Supercontig 13 [918385..920487] |
|  | PucGraTy3-24 | Broad Institute: Supercontig 24 [661664..663760] |
|  | PucGraTy3-25 | Broad Institute: Supercontig 237 [4676..6766] |
|  | PucGraTy3-26 | Broad Institute: Supercontig 11 [984672..986768] |
|  | PucGraTy3-27 | Broad Institute: Supercontig 19 [863604..865700] |
|  | PucGraTy3-28 | Broad Institute: Supercontig 1 [2361581..2363677] |
|  | PucGraTy3-29 | Broad Institute: Supercontig 10 [356450..358546] |
|  | PucGraTy3-30 | Broad Institute: Supercontig 27 [334994..337084] |
|  | PucGraTy3-31 | Broad Institute: Supercontig 114 [39619..41703] |
|  | PucGraTy3-32 | Broad Institute: Supercontig 27 [348993-351092] |
|  | PucGraTy3-33 | Broad Institute: Supercontig 15 [275792..278011] |
|  | PucGraTy3-34 | Broad Institute: Supercontig 12 [276028..278127] |
|  | PucGraTy3-35 | Broad Institute: Supercontig 5 [481534..483681] |
|  | PucGraTy3-36 | Broad Institute: Supercontig 78 [70492..72654] |
|  | PucGraTy3-37 | Broad Institute: Supercontig 8 [1468752..1470908] |
| *Batrachochytrium dendrobatidis* | BatDenTy3-1 | Broad Institute: Supercontig 13 [888518..890548] |
